# Path planning of industrial robots based on the adaptive field cooperative sampling algorithm

**DOI:** 10.3389/fnbot.2025.1574044

**Published:** 2025-11-13

**Authors:** Yongbo Zhuang, Sha Luo, Qingdang Li, Dianming Chu, Wenjuan Bai, Xintao Liu, Mingyuan Fan, Lv Wei

**Affiliations:** 1 College of Electromechanical Engineering, Qingdao University of Science and Technology, Shandong Province, China; 2 Shandong Gaomi Technician Institute, Shandong Province, China

**Keywords:** industrial robot, path planning, RRT, APF, AFCs

## Abstract

For the low efficiency and poor generalization ability of path planning algorithm of industrial robots, this work proposes an adaptive field co-sampling algorithm (AFCS). Firstly, the environment complexity function is proposed to make full use of environment information and improve its generalization ability of the traditional rapidly random search tree algorithm (RRT) algorithm. Then an optimal sampling strategy is proposed to make the improvement of the efficiency and optimal direction of RRT algorithm. Finally, this article designs a collaborative extension strategy, which introduces the improved artificial potential field algorithm (APF) into the traditional RRT algorithm to determine the new nodes, so as to improve the orientation and expansion efficiency of the algorithm. The proposed AFCS algorithm completes simulation experiments in two environments with different complexity. Compared with the traditional RRT, RRT* and tRRT algorithm, the results show that the AFCS algorithm has achieved great improvement in environmental adaptability, stability and efficiency. At last, ROKAE industrial robot is taken as the object to build a simulation environment for the path planning, which further verifies the practicability of the algorithm.

## 1 Introduction

Recently, the demand for intelligent robots is gradually increasing with the continuous development of artificial intelligence technology. Especially for industrial robots, path planning is a decisive factor to determine their safe operation. It refers to the autonomous planning of industrial robots in their configuration space to find a continuous non-collision smooth path between the initial pose and the target pose in order to reach the preset target pose when moving in the surrounding static and dynamic obstacle environment. Meanwhile, it must meet various constraints such as environmental, time and dynamic constraints of industrial robots (Wei and Ren, 2018). Different from that of mobile robots, it is more complex for industrial robots to realize path panning. It not only needs to consider constraints such as obstacles, but also involves the mutual transformation of joint space and configuration space. Therefore, the path planning algorithm of mobile robots is not completely applicable to the path planning of industrial robots. At present, there are three kinds of path planning algorithms of industrial robots: traditional obstacle avoidance planning method (Khatib, 1986; Hart et al., 1972), intelligent obstacle avoidance planning algorithm (Guan-Zheng and Huan, 2007; Kavraki et al., 1994), sampling-based obstacle avoidance planning algorithm (Lavalle, 1998; Karman and Frazzoli, 2011). Among them, the sample-based RRT algorithm (Lavalle, 1998) is the most applicable one in path planning algorithms of industrial robots, who has the characteristics of probability completeness and high dimensional space applicability (Lixing Liu et al., 2023). And it was also proved that the sample-based RRT algorithm owned the better efficiency and more smoother path than the intelligent obstacle avoidance planning algorithm for the industrial robots (Larsena, 2017). However, the traditional RRT algorithm has some problems such as redundant sampling, low efficiency and non-optimal path, which limit its generalization ability in complex environment (Jia et al., 2022). For these problems, research and improvement of RRT algorithm is a hot topic pursued by many scholars.

Among the variants of RRT algorithm, neither RRT algorithm, RRT* algorithm (Karman and Frazzoli, 2011) nor Informed RRT* algorithm (Gammell et al., 2014) fundamentally solve the randomness, low efficiency and optimality. Especially, in the sampling stage, redundant sampling brings a large number of branches, which not only leads to low efficiency, but also occupies a large amount of memory space. Therefore, based on the above algorithms, scholars have make some research on RRT algorithm from the aspects of sampling and expansion strategy. In terms of sampling strategies, the paper (Biao and Cao, 2021; Khan, 2020) adopted target paranoia to achieve “de-randomness.” But this strategy only reduced a small amount of the redundant sampling. Therefore, to further reducing the sampling space, Liu et al. (2020), Chi et al. (2022) and Ganesan and Natarajan (2021) set the sampling interval of the traditional RRT algorithm in different ways to make the search more efficient. On the aspect of the extension strategy, the paper (Zhang et al., 2019; Wang et al., 2020; Kang et al., 2016; Kang, 2019; Khan, 2020) fused the RRT algorithm with APF algorithm. Specifically, it used the attractive action to guide the production of new nodes and improve the search speed of the algorithm. In this process, although the target attraction is introduced, the repulsion of obstacles is not considered, so the role of the APF algorithm cannot be fully simulated. Wang et al. (2022), Kabutan and Nishida (2018) get new nodes by the guiding effect of target gravity and obstacle repulsion. It improved not only the goal orientation but also the obstacle avoidance ability. But with the disadvantages of unreachable target and local minima, the APF algorithm made the RRT algorithm unable to find new sampling points in some cases (Yin et al., 2018). Therefore, a path planning algorithm with strong stability, high efficiency and strong generalization ability is urgently needed for the characteristics of high latitude and complex collision detection process of industrial robots.

To get more improvement on the adaptability and stability of the path planning algorithm, an APCS algorithm is proposed, which combines the efficient search and optimization ability of the improved APF algorithm with the completeness of the RRT algorithm. And it fundamentally solves the problem of environment adaptability and redundant sampling. Contributions of this paper are summarized as follows:

- An environment complexity function is proposed to make path planning algorithm adapt to the environment;
- An optimal sampling strategy is proposed, which not only makes full use of environmental information, but also considers the optimality of sampling points;
- A field cooperative expansion strategy is proposed, in which the improved APF algorithm is proposed and guides the generation of new nodes;
- A new algorithm called AFCS is proposed,which not only enhances the environmental adaptability of industrial robots, but also improves sampling quality and scaling efficiency of the path planning algorithm. The whole algorithm provides ideas for improving the intelligence of the path planning algorithm.

The rest of this article is arranged as follows: The principle of path planning of robots is analyzed, and the principle of the traditional path planning algorithm is introduced in section 2. Then the AFCS algorithms is introduced in section 3. Section 4, summary and analysis.

## 2 Materials and models

For industrial robots, it is a prerequisite for path planning to satisfy constraints in terms of kinematics and dynamics. In this article, the relevant theories of industrial robot path planning are further studied based on the full study of the kinematics of industrial robots.

### 2.1 Kinematic model of manipulators

**Figure 1:**
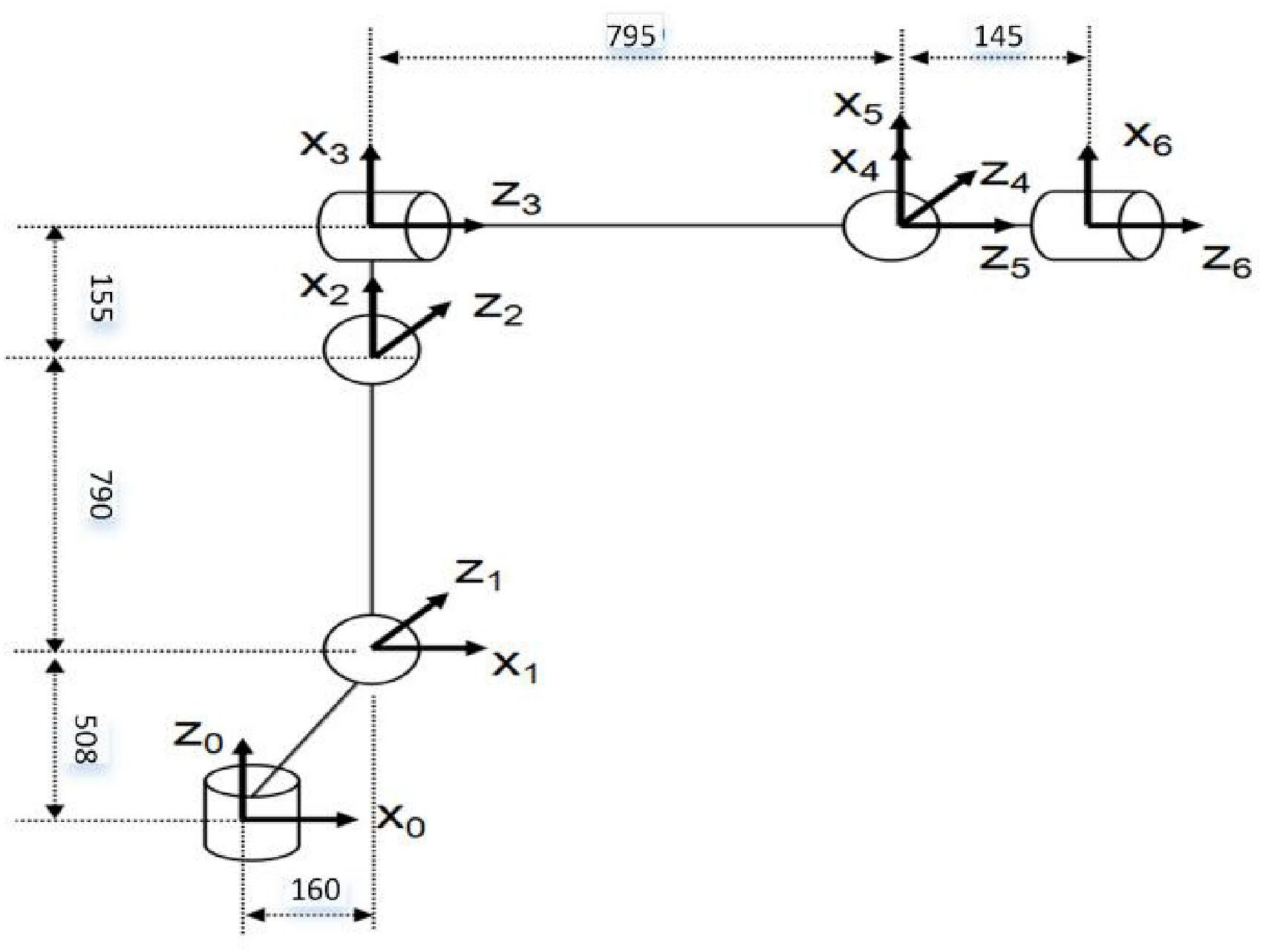
The coordinate system of ROKAE industrial robot.

Path planning of industrial robot needs mutual transformation in joint space and Cartesian space. So it involves the solution of inverse kinematics. And the accuracy of the inverse kinematics solution also certainly affects the accuracy of path planning of industrial robots. In this paper, ROKAE 6-DOF industrial robot is taken as the research object, and the coordinate system is established according to the DH model (Hartenberg et al., 1964) as shown in Figure 1.

Based on the homogeneous coordinate transformation matrix, the position and pose of the two adjacent coordinate systems i , i−1 of the industrial robot are obtained as follows:


$$\mathrm{Tii}-1=[\begin{array}{cccc} \cos\theta_{i} & -\sin\theta_{i}\cos\alpha_{i} & \sin\theta_{i}\sin\alpha_{i} & a_{i}\cos\theta_{i}\\ \sin\theta_{i} & \cos\theta_{i}\cos\alpha_{i} & -\cos\theta_{i}\sin\alpha_{i} & a_{i}\sin\theta_{i}\\ 0 & \sin\alpha_{i} & \cos\alpha_{i} & d_{i}\\ 0 & 0 & 0 & 1 \end{array}]\;\;\;$$ (1)

$\theta_{i}$ represents the angle between the X axes of adjacent coordinate systems, $\alpha_{i}$ represents the Angle between the Z axes of adjacent coordinate systems, $a_{i}$ is the length of the common perpendicular of the Z axis, $d_{i}$ is the distance between $a_{i}$ and $a_{i-1}$ .

According to the DH method, it is easy to get the forward kinematics equation of the industrial robot as follows:


$$T60=T10T21T32T43T54T65=[\begin{array}{cccc} n_{x} & o_{x} & a_{x} & p_{x}\\ n_{y} & o_{y} & a_{y} & p_{y}\\ n_{z} & o_{z} & a_{z} & p_{z}\\ 0 & 0 & 0 & 1 \end{array}]\;\;$$ (2)

Where, T1i−1 represents the homogeneous transformation matrix of adjacent coordinate systems. According to the principle of forward kinematics, the position and attitude of the industrial robot can be solved according to Equation 2. The inverse kinematics solution is the inverse process of the forward kinematics solution. In the path planning of industrial robots, forward and inverse kinematics can be calculated as needed. The DH parameters of the ROKAE industrial robot used in this paper are shown in Table 1.

**Table 1:** The DH parameters of the ROKAE industrial robot.

i	$d_{i}(\mathrm{mm})$	$\alpha_{i}(\deg)$	$a_{i}(\mathrm{mm})$	$\theta_{i}(\deg)$
1	508	−90	160	$\theta_{1}$
2	0	0	790	$\theta_{2}$
3	0	−90	155	$\theta_{3}$
4	795	90	0	$\theta_{4}$
5	0	−90	0	$\theta_{5}$
6	145	0	0	$\theta_{6}$

### 2.2 Path planning and model of environment

#### 2.2.1 Path planning

**Figure 2:**
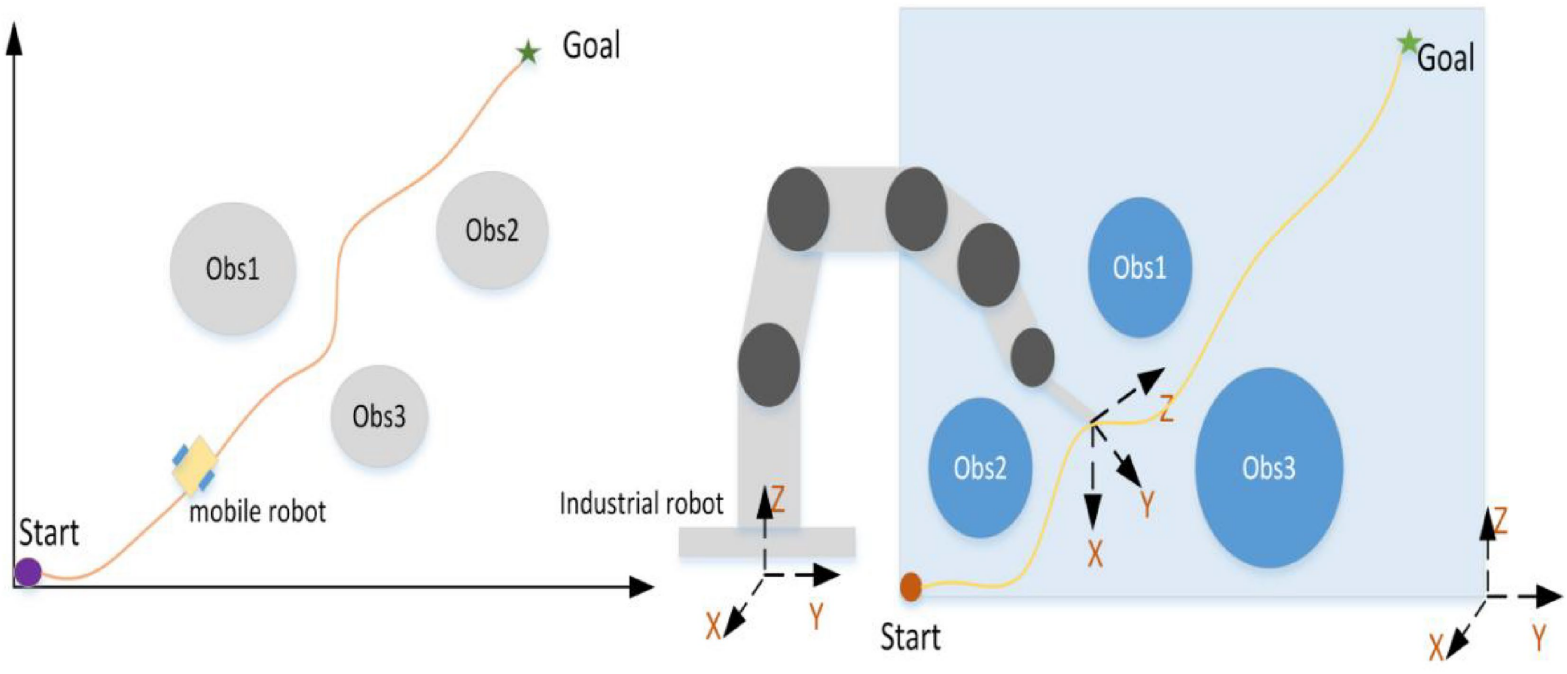
The schematic diagram of path planning of robots.

The core of path planning is planning, and its goal is to obtain a path that satisfies the conditions. The path is a continuous curve in the configuration space of robots. Specifically, path planning may be to plan a non-collision and shortest path for mobile robots in two-dimensional space. It also can plan a safe, non-collision or relatively optimized path for industrial robots (as shown in Figure 2). Jiang Xinsong, the father of Chinese robots, defines path planning as follow: The goal of path planning is to get a non-collision path in the environment with obstacles according to certain evaluation criteria (Hong et al., 2022). Deservedly, the different distribution of obstacles in the environment directly affects the planned path, and the target location determination is provided by the higher-level task decomposition module (Hong et al., 2022).

#### 2.2.2 Model of environment

**Figure 3:**
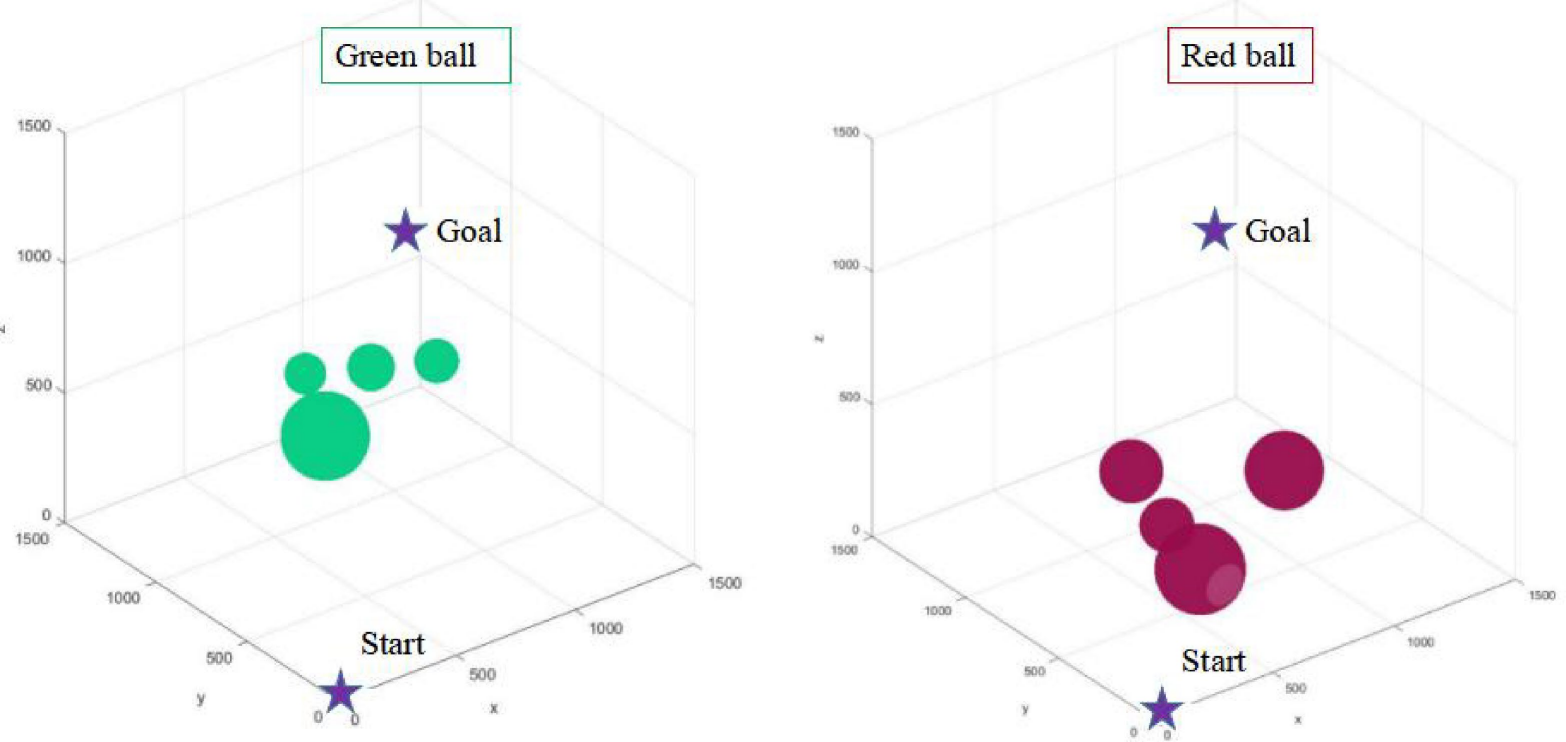
Two environmental maps with different complexity.

In traditional path planning, environment modeling is the first step of path planning in order to better satisfy configuration space constraints. However, it is more and more difficult to model the environment in the more complex scenario. The complexity of environment modeling is an important problem that restricts the path planning of robots. This article puts forward the environmental complexity function to counter the difficulty of environment modeling. This strategy realizes the prediction of the environment where the path planning is located, and lays a theoretical foundation for path planning algorithm to take full advantage of the environmental information. This paper designs two kinds of environment maps (as shown in Figure 3), in which the superiority of path planning algorithm can be better reflected.

As shown in Figure 3, the number of obstacles in two maps is the same, but the complexity of the environment is different because of the different distribution of obstacles. The pixel size of the two maps is1500*1500. In this paper, the starting point position coordinate of path planning is (0, 0, 0), and the target point coordinate is set to (1000, 1000, 1000).

### 2.3 Path planning algorithm

The sampling-based RRT algorithm and APF algorithm are the most widely used in path planning of industrial robots. By fully studying the traditional APF algorithm and RRT algorithm, this article summarizes the advantages and disadvantages of the two methods. At the same time, this article makes same improvement of the traditional algorithms in view of their shortcomings, and a new AFCS algorithm with strong environment adaptability is proposed.

#### 2.3.1 RRT algorithm

**Figure 4:**
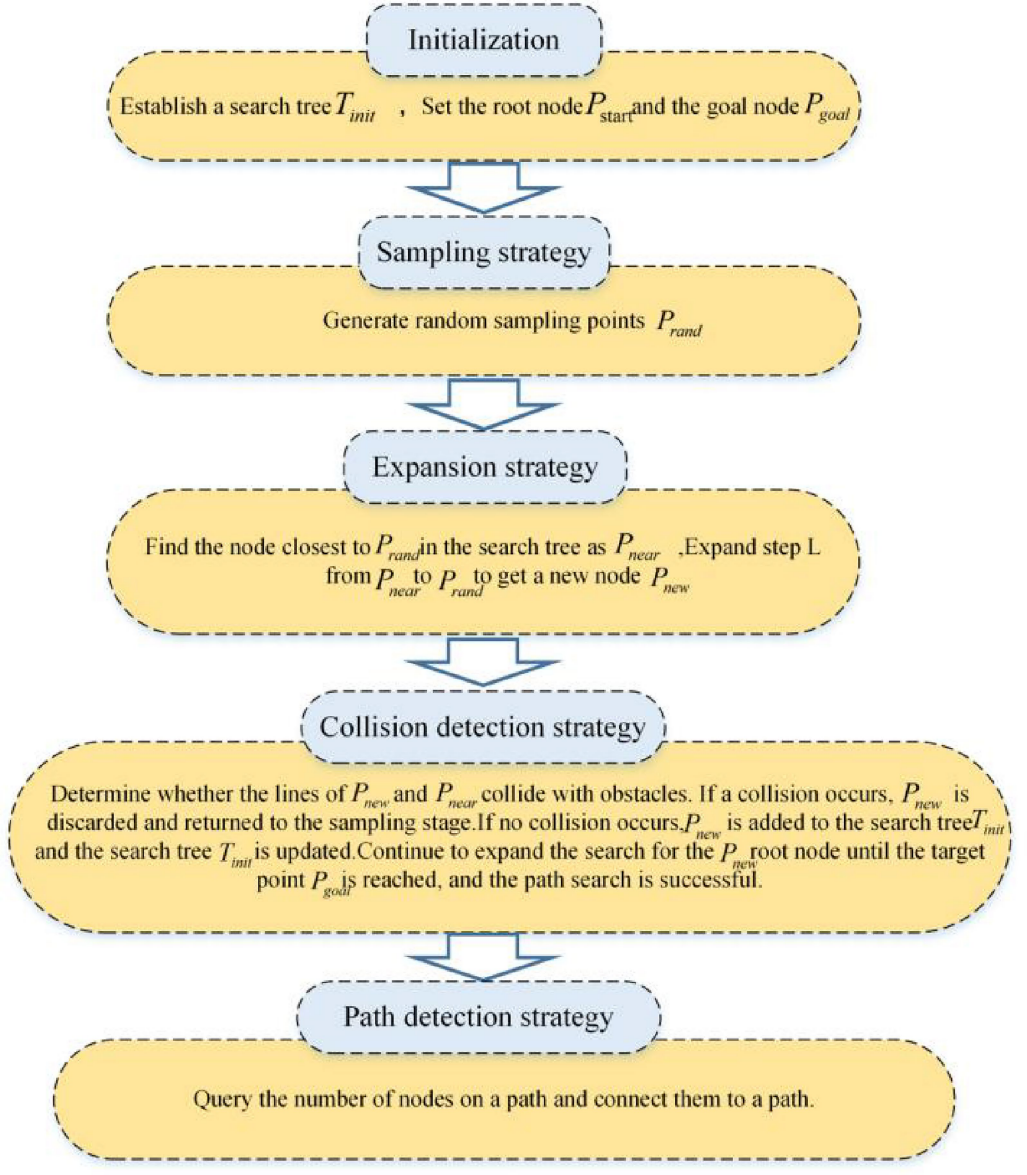
The implementation of the RRT algorithm.

**Figure 5:**
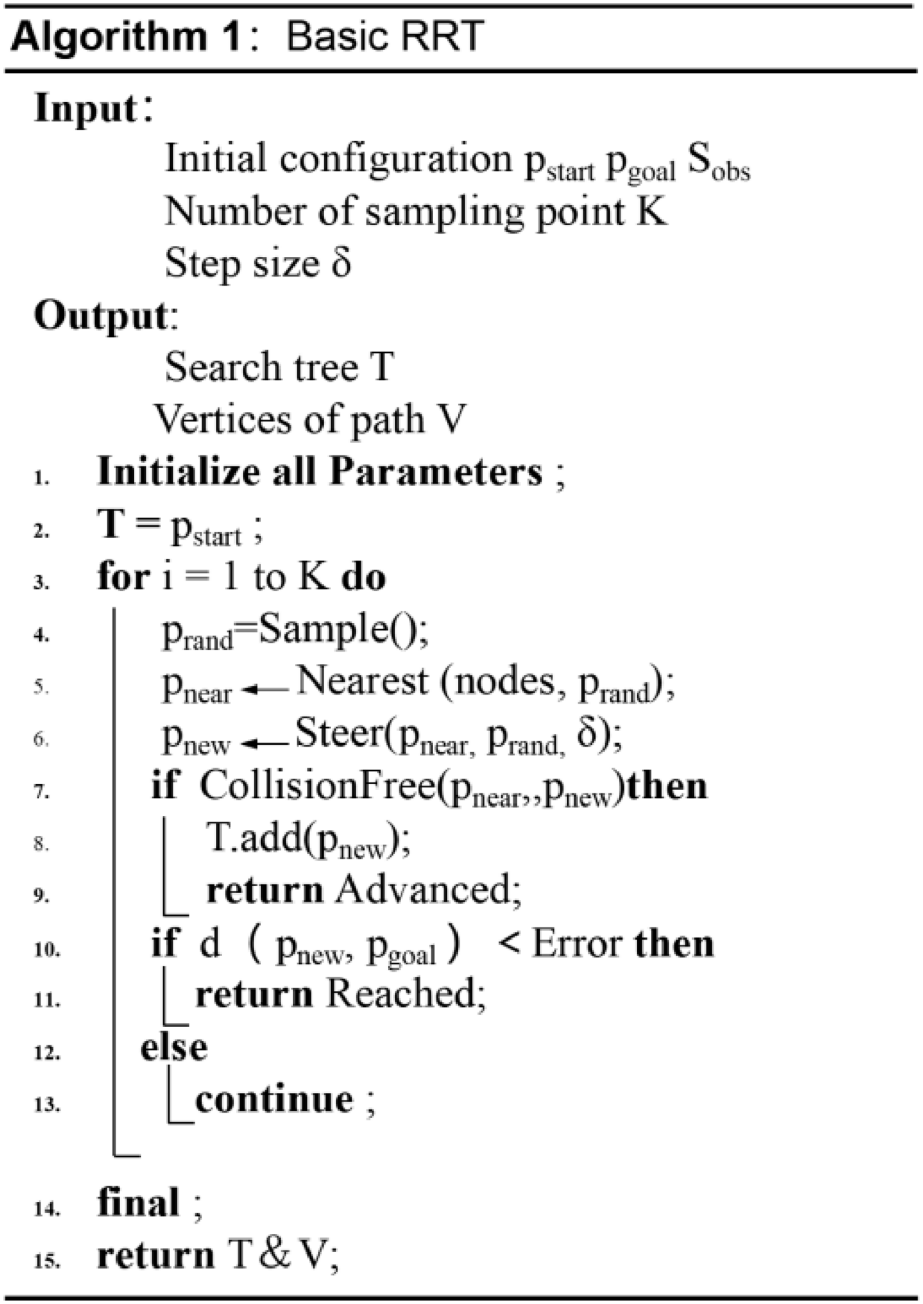
The pseudo-code of the traditional RRT algorithm.

There is a path search algorithm called the RRT algorithm, which expands by random sampling. It establishes the search tree from the starting point. Subsequently, it undergoes random sampling, expands new nodes, avoids obstacles, and finally finds an optimal path. Figure 4 shows the implementation process of the traditional RRT algorithm, and its pseudo-code is shown in Figure 5.

The core of traditional RRT algorithm can be divided into four stages: sampling, expansion, collision detection and path query. There are three important factors that lead to the low efficiency and poor applicability of RRT algorithm, which are the redundant sampling in the sampling phase, the way and efficiency of generating new nodes in the expansion phase, and the amount of calculation in collision detection (Junxiang and Wang, 2021). Especially in the sampling stage, the redundant sampling not only has strong randomness, but also does not consider the optimality of sampling. Moreover, in the measurement connection stage, the generation of new nodes is neither guided nor considering the influence of obstacles, so that the calculation of collision detection of the RRT algorithm is very large. Generally, the randomness and efficiency of the RRT algorithm are important factors affecting its development. Therefore, this paper proposes an efficient RRT algorithm to solve the strong randomness and low efficiency.

#### 2.3.2 APF algorithm

The APF algorithm is a simple and effective local path planning algorithm. It constructed by Dr. Oussama Khatib who introduced the concept of “field” in traditional mechanics (Fan et al., 2020; Rostami, 2019). The potential field includes the attractive and repulsive potential field, which are formed by the target and the obstacle, respectively. The agent moves along the resultant force of the attractive and repulsive forces (Yuan et al., 2021). The attractive and repulsive potential field functions are modeled as follows


$$U_{\mathrm{att}}(P)=\frac{1}{2}k_{t}\|P-P_{\text{goal}}\|$$ (3)

$$U_{\mathrm{rep}}(P)=\{\begin{array}{cc} 0 & \mathrm{dP}\|-P_{\mathrm{obs}}\|\;\geq D_{2}\\ \frac{k_{r}}{2}(\frac{1}{\left\|P-P_{\mathrm{obs}}\right\|}-\frac{1}{D_{2}}) & \mathrm{dP}-\|P_{\mathrm{obs}}\|\;<D_{2} \end{array}$$ (4)

Where, $U_{\mathrm{att}}(P)$ denotes the attractive potential field of the node $P,k_{t}$ is the attractive coefficient. $\left\|P-P_{\text{goal}}\right\|$ denotes the distance between two nodes P and $P_{\text{goal}}$ ; The repulsive potential field generated by the node P is expressed as $U_{\mathrm{rep}}(P),k_{r}$ is the repulsion coefficient; $\left\|P-P_{\mathrm{obs}}\right\|$ denotes the distance between two nodes P and $P_{\mathrm{obs}}$ _;_ $D_{2}$ indicates the influence threshold of an obstacle; When $\|P-P_{\mathrm{obs}}\|\;\geq D_{2}$ _,_ the node is not affected by obstacles; When $P-P_{\text{goal}}<D_{2}$ , the node is affected by the repulsive potential field of the obstacle.

The model of the attractive and repulsive forces are shown as follow:


$$F_{\mathrm{att}}(P)=-\Delta U_{\mathrm{aat}}(P)=k_{t}\|P-P_{\text{goal}}\|$$ (5)

$$\begin{array}{l} F_{\mathrm{rep}}(P)=-\Delta U_{\mathrm{rep}}(P)=\\ \left\{ \begin{array}{cc} 0 & d\|P-P_{\mathrm{obs}}\|\geq D_{2}\\ -k_{r}(\frac{1}{\left\|P-P_{\mathrm{obs}}\right\|}-\frac{1}{D_{2}}) & d\|P-P_{\mathrm{obs}}\|<D_{2} \end{array}\right. \end{array}$$ (6)

The resultant force of attraction and repulsion is shown as follow:


$$F_{\text{total}}(P)=F_{\mathrm{att}}(P)+F_{\mathrm{rep}}(P)$$ (7)

**Figure 6:**
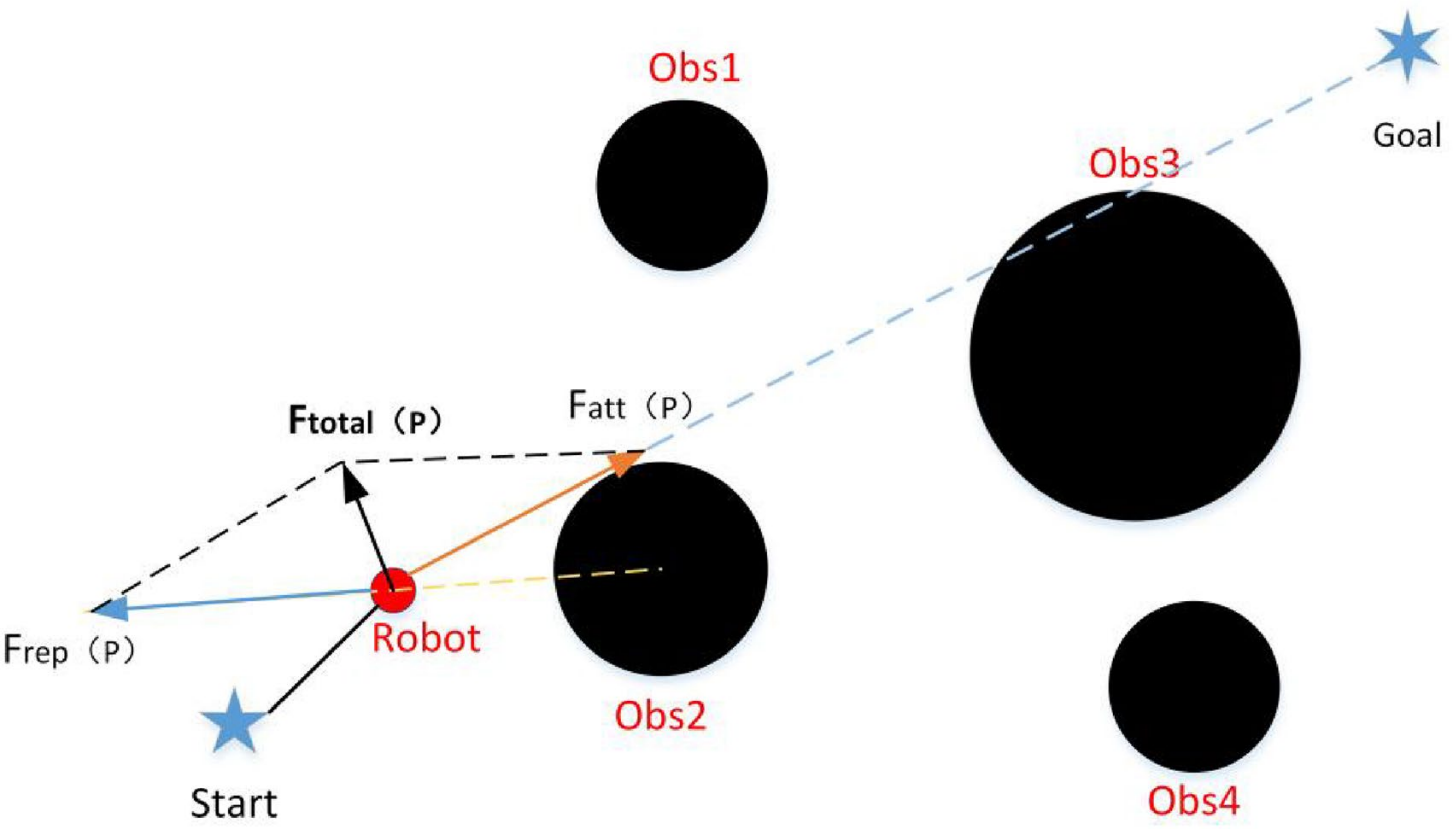
The schematic diagram of path planning of the APF algorithm.

The agent realizes obstacle avoidance path planning under the guidance of the resultant force, and its schematic diagram is shown in Figure 6.

On the basis of the principle of the APF algorithm, it can be known that the magnitude of the attraction and repulsion force of the agent are related to the corresponding distance. So its main disadvantages are the inaccessible target and local minimum (Wang et al., 2022). Otherwise, the essence of the APF algorithm is a control method, and its path is generated by the control quantity in real time. Therefore, its real-time performance is more strong. For the shortcomings of the APF algorithm, this paper proposes an improved APF algorithm, and introduces it into the traditional RRT algorithm to improve its real-time performance.

### 2.4 The adaptive field cooperative sampling algorithm

On the principle of path planning algorithm, this paper innovatively put forward an adaptive field cooperative sampling algorithm. Firstly, the environment complexity function is proposed for the difficulty of environment modeling. Then, an improved APF algorithm is proposed to solve the inaccessible target and local minimum. Finally, based on the framework of the traditional RRT algorithm, an optimal sampling strategy is proposed, and the AFCS algorithm is obtained by introducing the environment complexity function and the improved APF algorithm into the RRT algorithm.

#### 2.4.1 Environment complexity function

Aiming at the complex of environment modeling, this article designs the environment complexity function *S* based on the relative position of obstacles in the environment. Then it is introduced into the traditional RRT algorithm. This operation enables the algorithm to intelligently adjust the iteration time instead of manually adjusting the iteration parameters according to the complexity of the environment. And it also improves its environmental adaptability. On the basis of the volume ratio of obstacles, this article introduces the distance between obstacles to further distinguish the complexity, so as to make the path planning algorithm suitable for environments with different complexity. The environment complexity function is as follows:


$$S=\lambda_{1}\frac{n_{1}V_{\mathrm{ob}s_{1}}e^{10}}{VL^{3}}$$ (8)

Where, S represents the environment complexity. $n_{1}$ respectively indicates the number of static in the environment (Urain et al., 2023). $V_{\mathrm{obs}1}$ represents the volume of static obstacles. V represents the total volume. Only one class of static obstacle cases is studied here, so $\lambda_{1}=1$ .

According to the proposed environment complexity function, the environment complexity of the two maps in Figure 4 can be, respectively, calculated as: 2301, 1809. Although the number of obstacles in the two maps is the same, the relative position of obstacles is different. In the green sphere environment, the minimum distance between obstacles is 697 mm. In the red sphere environment, the minimum distance between obstacles is 883 mm. The green sphere environment is relatively complex. Therefore, the design of the environment complexity function is reasonable.

#### 2.4.2 Optimal sampling strategy

For the RRT algorithm and its variants, the ideal sampling procedure is one that reduces redundant sampling while keeping the sampling point on or near the optimal path as much as possible. Based on the above ideas, this article proposes an optimal sampling strategy to solve redundant sampling and poor quality of sampling points in the sampling stage. The idea of this strategy is to randomly generate multiple sampling points at the same time, and then determine the sampling quality function based on the density of obstacles around the sampling points and the smoothness of the path. In this way, random sampling points with optimal quality can be obtained. The sampling quality function is modeled as follows:


$$M_{P_{\text{randi}}}={w_{1}}^{*}\rho_{P_{\text{randi}}}+{w_{2}}^{*}\sigma_{P_{\text{randi}}}$$ (9)

$$P_{\text{randi}}=\frac{\max\|P_{\text{randi}}-P_{\mathrm{obs}}\|}{A}$$ (10)

$$\sigma_{\text{randi}}=\frac{∠P_{\text{randi}}P_{\text{nearest}}P_{k-1}}{180^{\circ }}$$ (11)

Where, $M_{P_{\text{randi}}}$ represents the quality function of the *i* random sampling point $P_{\text{randi}}$ . $\rho_{P_{\text{randi}}}$ is the density of obstacles around the random sampling point $P_{\text{randi}}$ . $\sigma_{P_{\text{randi}}}$ represents the smoothness of the random sampling point $P_{\text{randi}}$ . $\left\|P_{\text{randi}}-P_{\mathrm{obs}}\right\|$ represents the distance between the random sampling point $P_{\text{randi}}$ and the nearest obstacle $P_{\mathrm{obs}}∠P_{\text{randi}}P_{\text{nearest}}P_{k-1}$ represents the angle between the line $P_{\text{randi}}\;P_{\text{nearest}}$ and $P_{\text{nearest}}P_{k-1}\;w_{1},w_{2}$ represent the influence factors of obstacle density and smoothness of sampling points.

**Figure 7:**
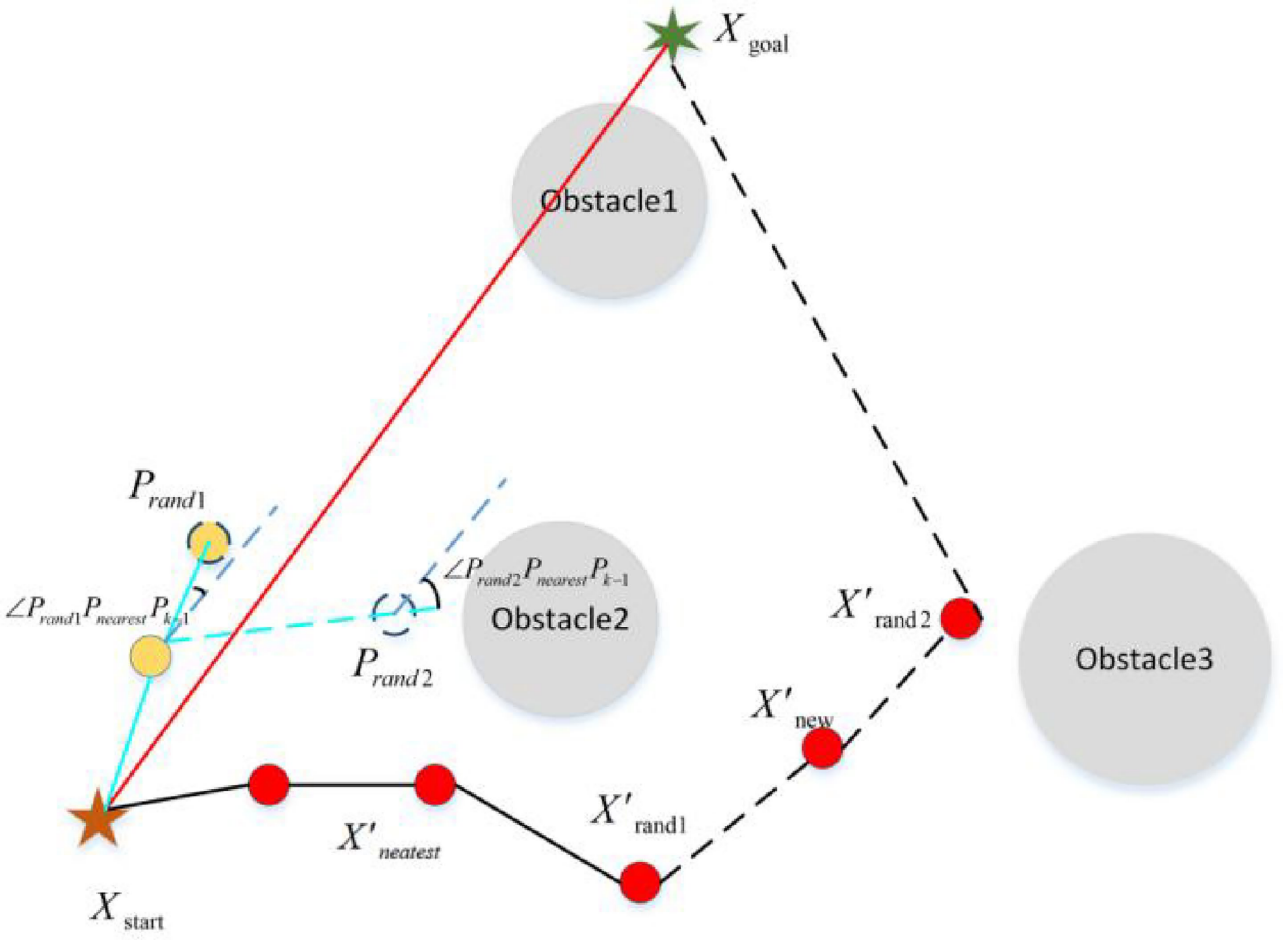
The schematic diagram of optimal sampling process (the blue path represents the optimal sampling process, and the black path represents the path generated by the random sampling process).

The schematic diagram of optimal sampling process is shown in Figure 7.

#### 2.4.3 Field cooperative expansion strategy

##### 2.4.3.1 The improved APF algorithm

For the problem of the inaccessible target and local minimum, this article, respectively, introduces the distance between the target point and the robot into the attractive potential field and the repulsive potential field function of the traditional APF algorithm. Based on papers (Ahmadi et al., 2022; Guo et al., 2022), the attractive and repulsive potential field models proposed in this paper are shown as follow:


$$U_{\mathrm{att}}(P)=\{\begin{array}{cc} \frac{k_{t}\|P-P_{\text{goal}}\|}{2} & d\|P-P_{\text{goal}}\;\|\;\leq D_{1}\\ D_{1}k_{t}\|P-P_{\text{goal}}\|-\frac{k_{t}D_{1}^{2}}{2} & d\|P-P_{\text{goal}}\;\|\;>D_{1} \end{array}$$ (12)

$$\begin{array}{l} U_{\mathrm{rep}}(P)=\\ \left\{ \begin{array}{cc} 0 & d\|P-P_{\mathrm{obs}}\;\|\;\geq D_{2}\\ \frac{k_{r}}{2}(\frac{1}{\left\|P-P_{\mathrm{obs}}\right\|}-\frac{1}{D_{2}})-\frac{1}{2}\sigma \|P-P_{\text{goal}}\| & d\|P-P_{\mathrm{obs}}\|\;<D_{2} \end{array}\right. \end{array}$$ (13)

Where, $U_{\mathrm{att}}(P)$ represents the attractive potential field, $k_{t}$ is the attractive coefficient. $\left\|P-P_{\text{goal}}\right\|$ denotes the distance between two nodes *P*and t $P_{\text{goal}}$ _._ $D_{1}$ represents the threshold for the node to reach the target point. When $\|P-P_{\text{goal}}\|\;\leq D_{1}$ , the robot approaches the target point at a faster speed. Otherwise, the robot approaches the target point at a slower speed. $U_{\mathrm{rep}}(P)$ represents the repulsive potential field. $k_{r}$ is the repulsion coefficient. $\left\|P-P_{\mathrm{obs}}\right\|$ denotes the distance between two nodes *P* and $P_{\mathrm{obs}}$ _;_ $D_{2}$ indicates the influence threshold of an obstacle. σ represents the distance influence factor. When $\|P-P_{\mathrm{obs}}\|\;\geq D_{2}$ _,_ the node is not affected by obstacles; When $\|P-P_{\text{goal}}\|<D_{2}$ , the repulsive potential field of the obstacle dose not work.

The attractive and repulsive function models are shown as follows:


$$F_{\mathrm{att}}(P)=\{\begin{array}{cc} k_{t}\|P-P_{\text{goal}}\| & d\|P-P_{\text{goal}}\|\leq D_{1}\\ D_{1}\cdot k_{t}\cdot \|P-P_{\text{goal}}\|-k_{t}\cdot D_{1} & d\|P-P_{\text{goal}}\|>D_{1} \end{array}$$ (14)

$$\begin{array}{l} F_{\mathrm{rep}}(P)=\\ \left\{ \begin{array}{cc} 0 & d\|P-P_{\mathrm{obs}}\|\geq D_{2}\\ -k_{r}(\frac{1}{\left\|P-P_{\mathrm{obs}}\right\|}-\frac{1}{D_{2}})+\sigma \|P-P_{\text{goal}}\| & d\|P-P_{\mathrm{obs}}\|<D_{2} \end{array}\right. \end{array}$$ (15)

In this paper, the field cooperative expansion strategy is to introduce the improved APF algorithm into the traditional RRT algorithm. The position and orientation of the new nodes are calculated according to the improved APF algorithm. The new node is calculated as follows:


$$P_{\mathrm{new}}=P_{\text{nearest}}+\beta_{1}P_{\mathrm{rand}}+\beta_{2}\frac{F_{\text{total}}(P)}{\left\|F_{\text{total}}(P)\right\|}$$ (16)

$$F_{\text{total}}(P)=F_{\mathrm{att}}(P)+F_{\mathrm{rep}}(P)$$ (17)

Where, $P_{\mathrm{new}}$ is the new node. $P_{\mathrm{rand}}$ represents the random sampling point, whose nearest point on the random tree is expressed as $P_{\text{nearest}}$ . $\beta_{1},\beta_{2}$ represents the influence factors of the random node and target gravity on the new node. $F_{\text{total}}(P)$ represents the resultant force of the potential field. $F_{\mathrm{att}}(P)$ represents the sum of the attractive forces of the random node and the target node on the new node. $F_{\mathrm{rep}}(P)$ represents the sum of the repulsive forces of obstacles on new nodes.

**Figure 8:**
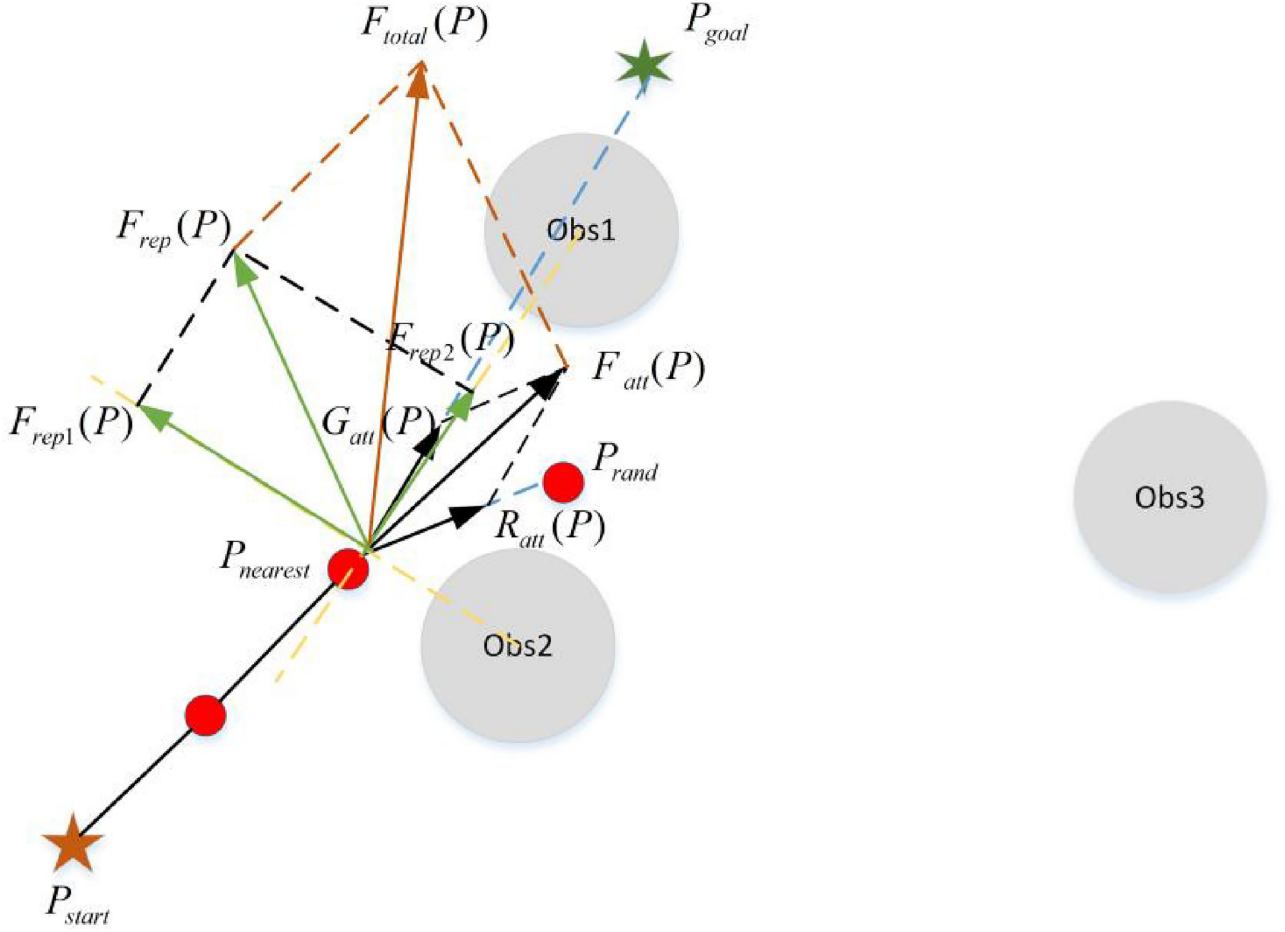
The schematic diagram of field cooperative expansion strategy.

The schematic diagram of field cooperative expansion strategy is shown in Figure 8.

#### 2.4.4 The adaptive field cooperative sampling algorithm

**Figure 9:**
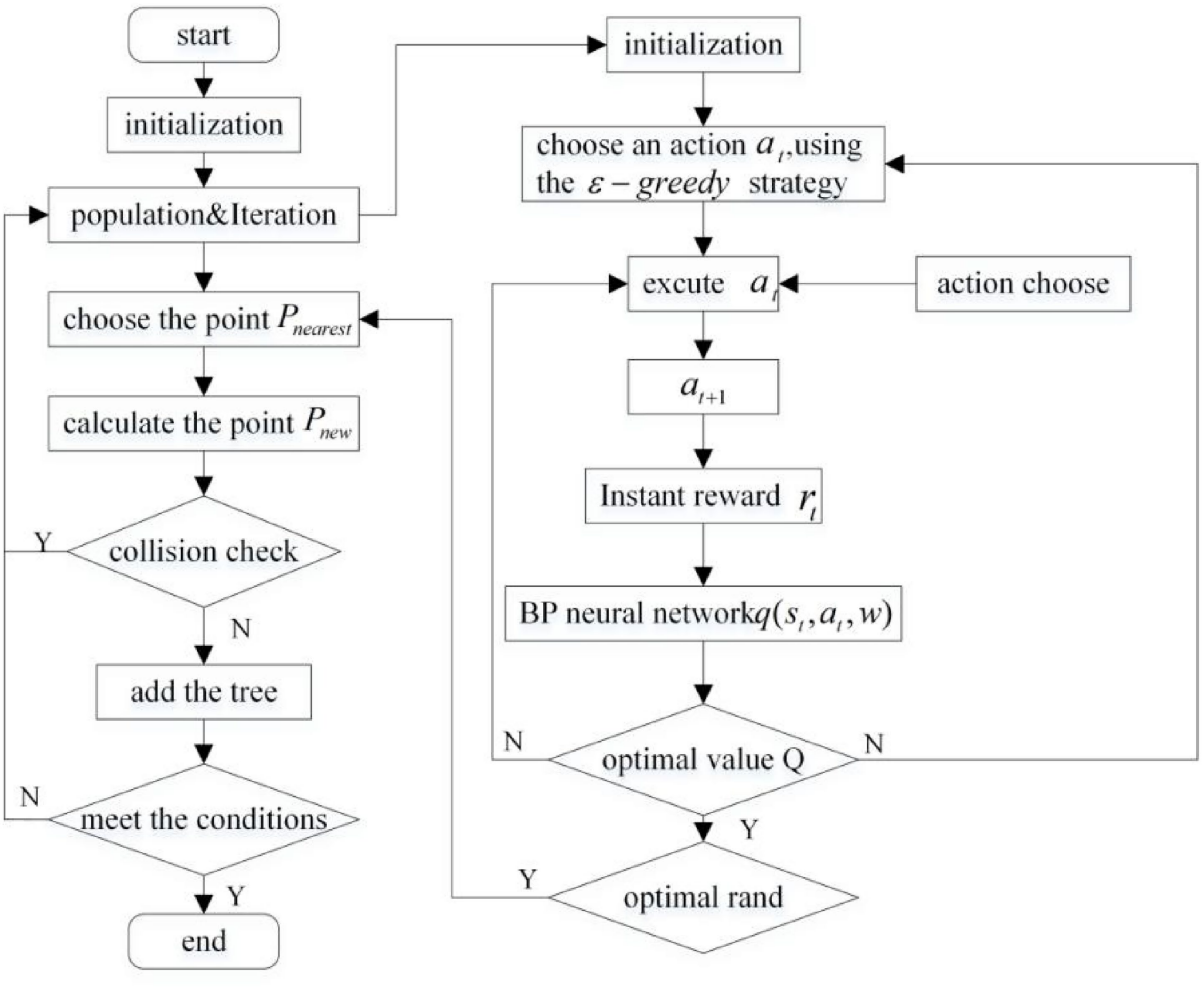
The flow chart of the AFCS algorithm.

Based on the above innovative strategy, an adaptive field co-sampling algorithm is proposed in this paper. It not only avoids complex environment modeling, but also makes full use of environment information. Moreover it solves the redundant sampling and low efficiency of traditional RRT algorithm by the optimal sampling strategy and the introduction of improved APF algorithm. The flow chart of the AFCS algorithm is shown in Figure 9.

**Figure 10:**
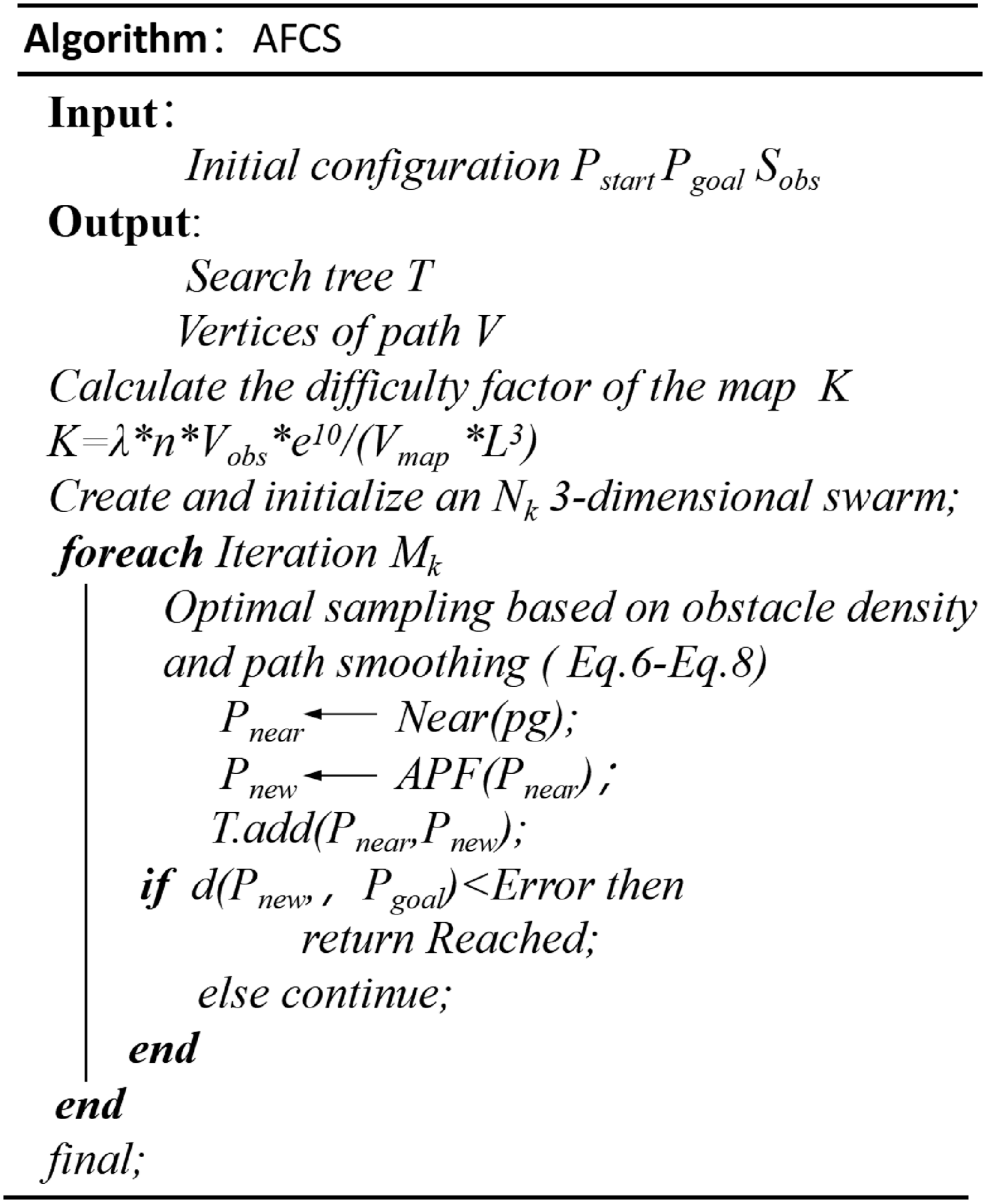
The pseudo-code of the AFCS algorithm.

The pseudo-code of the AFCS algorithm is shown in Figure 10.

## 3 Experiments and results

Simulation experiment is an important way to show the superiority of the algorithm. The simulation experiment in this paper is carried out in two aspects: algorithm simulation and industrial robot simulation.

In terms of algorithm simulation, two environments with different complexity are used to carry out simulation experiments to verify the environmental adaptability of the algorithm. At the same time, this paper verifies the effectiveness and practicability of the AFCS algorithm by analyzing the experimental results with the traditional RRT algorithm, RRT* algorithm and tRRT algorithm from three aspects: planning time, path cost and the number of path points.

In the aspect of industrial robot simulation, this paper mainly integrates the AFCS algorithm with industrial robot simulation to verify its effectiveness and practicability.

**Figure 11:**
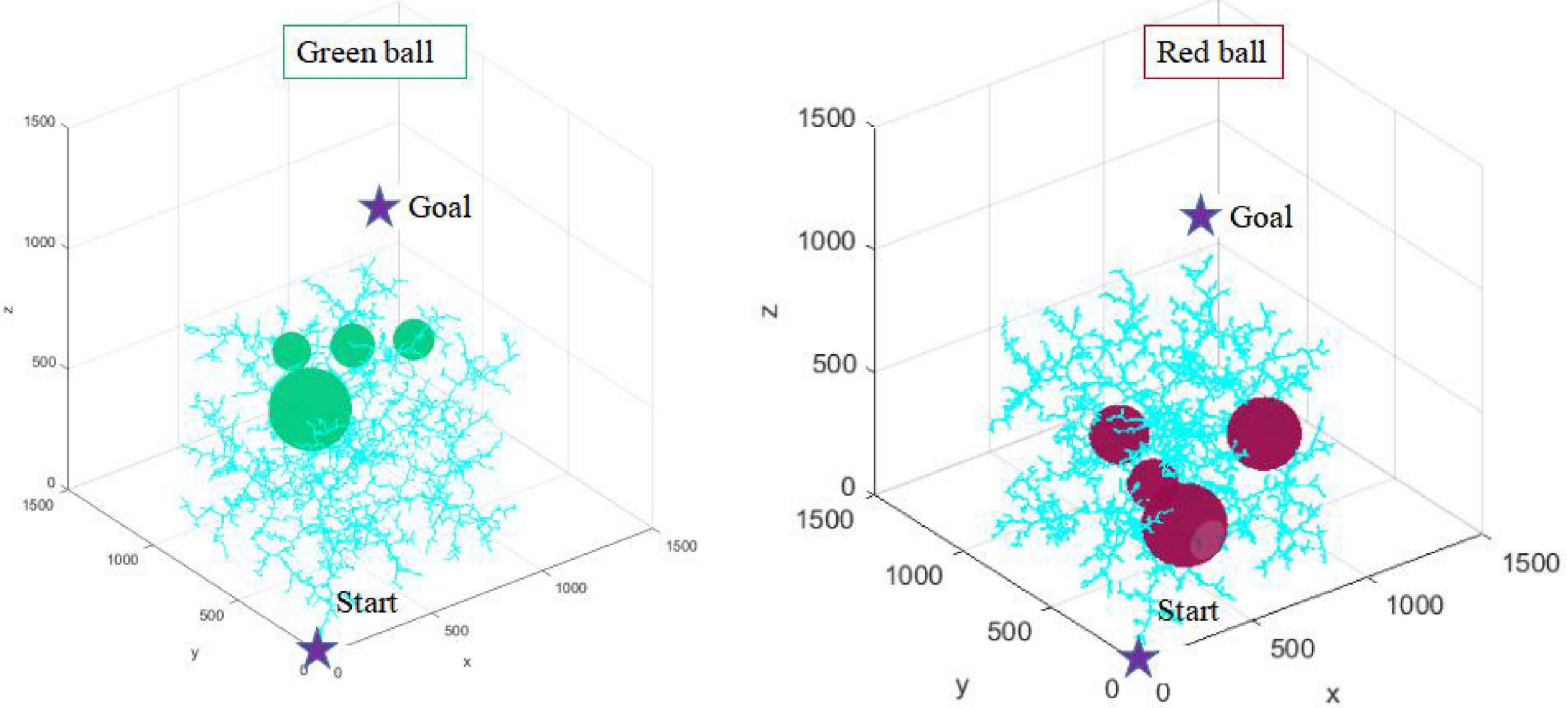
The simulation results of the RRT algorithm in two environments.

**Figure 12:**
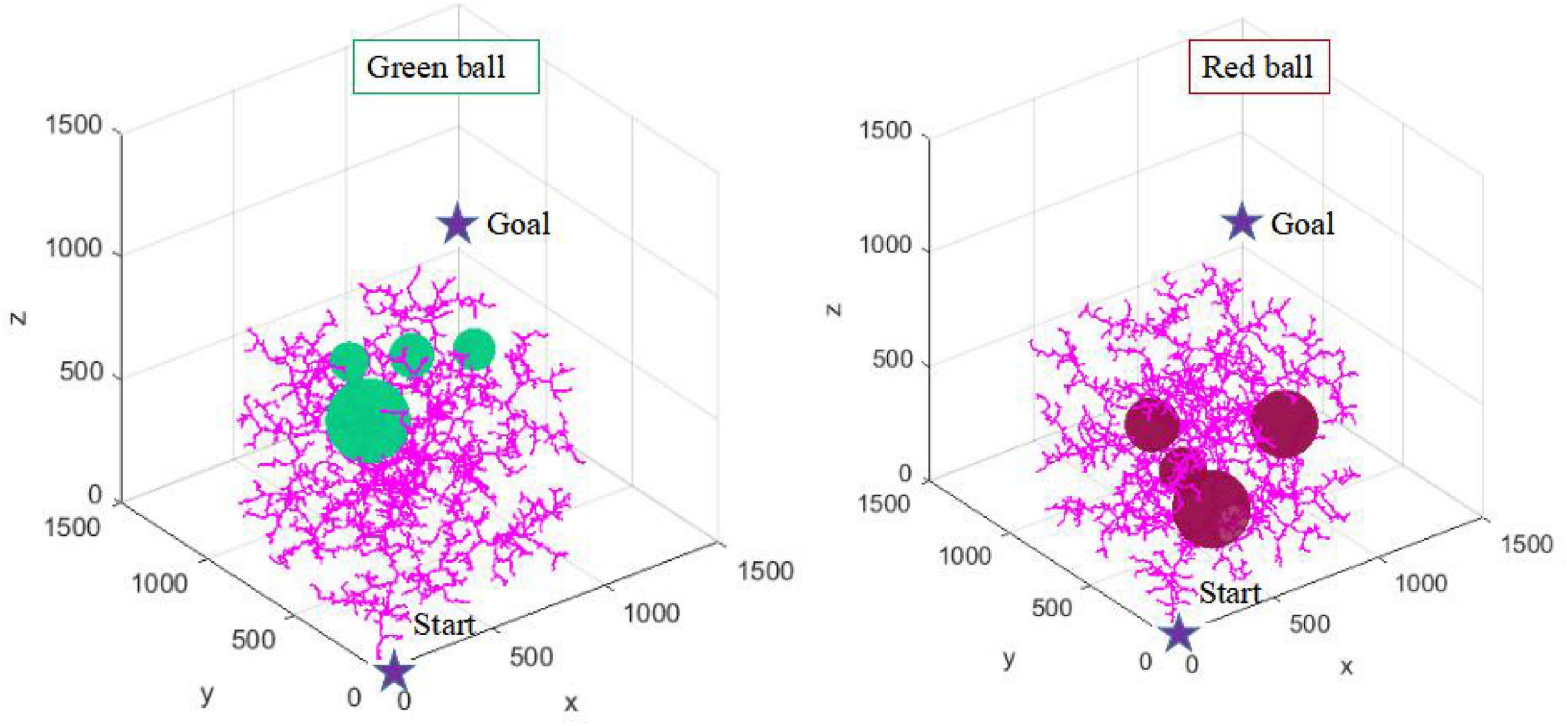
The simulation results of the RRT* algorithm in two environments.

In two maps with different complexity, this article conducts multiple simulation experiments with the same number of iterations and step size, respectively. The multiple operation results of the traditional RRT algorithm and RRT* algorithm are shown in Figures 11, 12.

In Figures 11, 12, the RRT algorithm and RRT* algorithm fail the path planning due to the large amount of redundant sampling in the tow environments. Although both RRT and RRT* algorithms are probabilistic complete, such a time-consuming strategy is not desirable in practice. Therefore, this article focuses on the comparison and analysis of simulation results between the AFCS algorithm and the most representative tRRT algorithm.

### 3.1 Experiment of algorithm simulation

#### 3.1.1 Analysis of environmental adaptability

**Figure 13:**
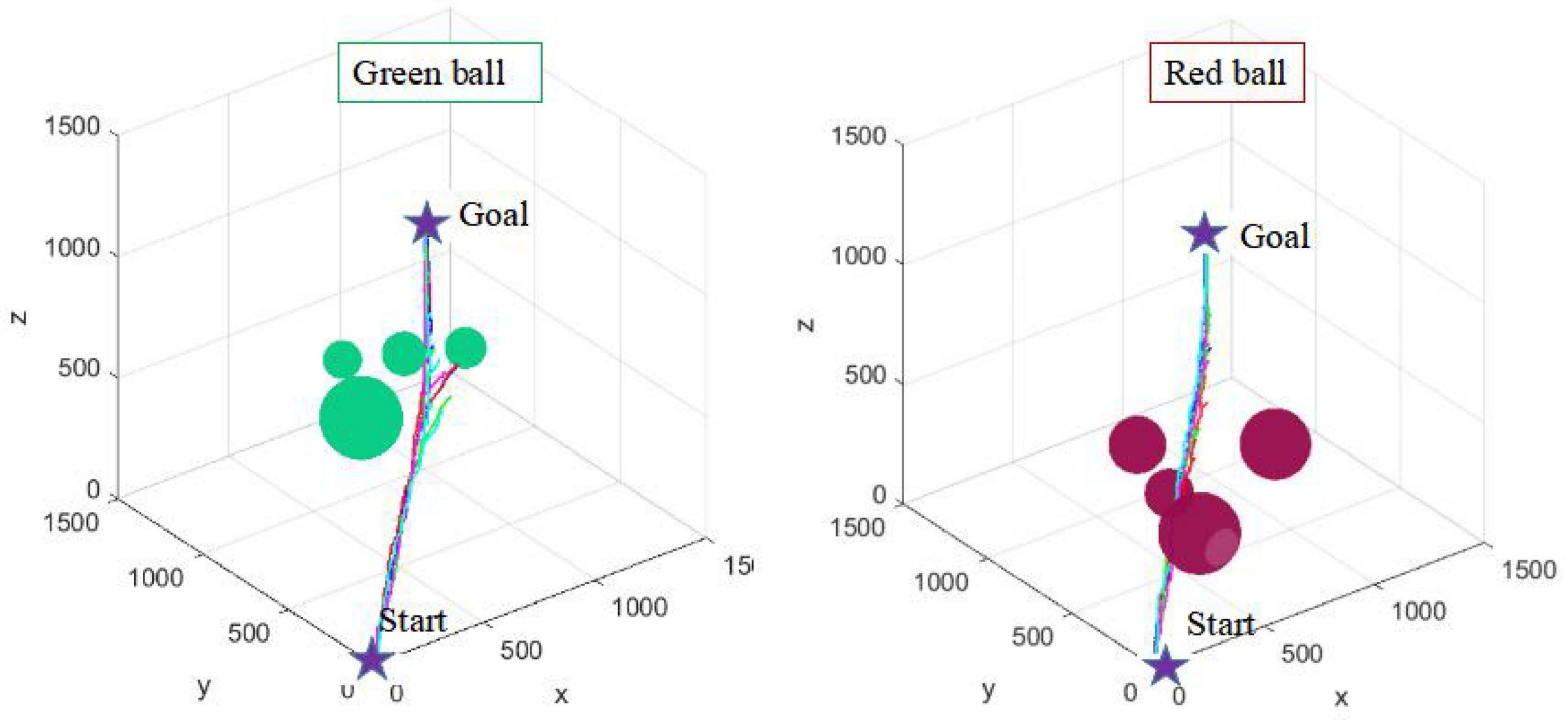
Five consecutive simulation results of the tRRT algorithm.

**Figure 14:**
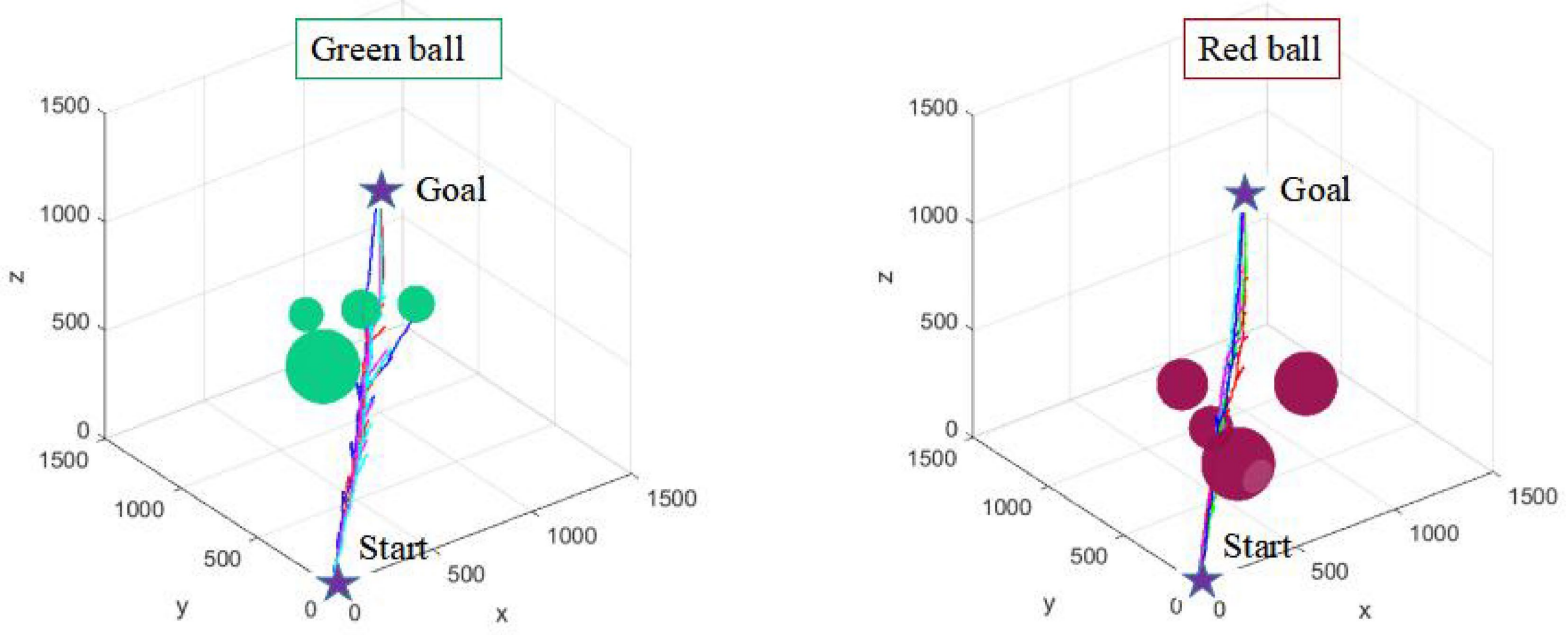
Five consecutive simulation results of the AFCS algorithm.

Environmental adaptability is an embodiment of the intelligence of path planning algorithm. It is also an important direction to improve the intelligence of industrial robots. Five consecutive simulation experiments are carried out in two environments with different complexity (as shown in Figures 13, 14) to verify the environmental adaptability of the algorithm. It should be noted that the number of iterations of the AFCS algorithm is related to the environment complexity function without manual mediation. The iteration and step size of the tRRT algorithm are the same.

It can be seen from Figures 13, 14 that the results of the AFCS algorithm are more stable. And the branches of the path obtained by the AFCS algorithm are less. But when the environment of the tRRT algorithm changes (from red sphere environment to green sphere environment), the branches of its path becomes more and more. Therefore, the proposed AFCS algorithm has better adaptability to the environment and higher stability.

#### 3.1.2 Analysis of efficiency

Time is an important reflection of efficiency. In this paper, the planning time of the algorithm in the same environment is taken as one of the criteria to measure the efficiency of the algorithm. The AFCS algorithm and tRRT algorithm are run 10 times in two environments with different complexity to obtain the average planning time of the algorithm (as shown in Table 2).

**Table 2:** The planning time of the AFCS algorithm and tRRT algorithm.

i	Green Ball		Red Ball	
	AFCS	tRRT	AFCS	tRRT
1	4.8414	7.4142	4.7543	4.4731
2	4.5426	5.9894	4.3211	4.9958
3	4.5295	5.2871	4.4687	4.3985
4	3.8931	6.4891	4.3451	4.6828
5	4.6952	5.3633	4.2883	4.1987
AVE	4.5004	6.1086	4.4355	4.5498

According to the data in Table 2, in the environment with different complexity, the planning time of the AFCS algorithm remains stable and does not float with the change of environment. In contrast, the efficiency of the tRRT algorithm fluctuates by about 27% as the complexity of the environment changes. This fluctuation of operating efficiency will affect the expansion of application scenarios of the algorithm. On the other hand, in the environment with the same complexity, the AFCS algorithm has shorter planning time and higher stability than the tRRT algorithm.

This paper runs the AFCS algorithm and the tRRT algorithm for 20 times, respectively, in the green sphere environment to further verify the stability of the AFCS algorithm. The path planning time of the two algorithms is shown in Table 3.

**Table 3:** The running time of the AFCS algorithm and tRRT algorithm.

AFCS	No	1	2	3	4	5	6	7	8	9	10
	Time (s)	5.593	47.252	5.336	26.35	5.739	19.945	6.995	40.209.506	12.134	19.228
	No	11	12	13	14	15	16	17	18	19	20
	Time (s)	16.58	18.991	7.135	23.16	4.899	32.178	36.55	15.46	18.733	4.976
tRRT	No	1	2	3	4	5	6	7	8	9	10
	Time (s)	3.804	3.9724	4.1898	4.753	4.298	4.2431	4.4561	4.5607	4.569	4.145
	No	11	12	13	14	15	16	17	18	19	20
	Time (s)	4.235	4.1579	4.3760	4.409	4.3345	4.4708	4.4809	4.3547	4.6079	5.1245

In Table 3, the planning time of 20 times of the AFCS algorithm is stable between 3.904 s–5.2919 s, and the planning time fluctuates little in the green sphere environment. However, the 20 times path planning time of tRRT algorithm fluctuates from 4.4478 s to 46.7573 s. Therefore, the AFCS algorithm not only has high planning efficiency, but also has good stability.

#### 3.1.3 Analysis of path quality

**Figure 15:**
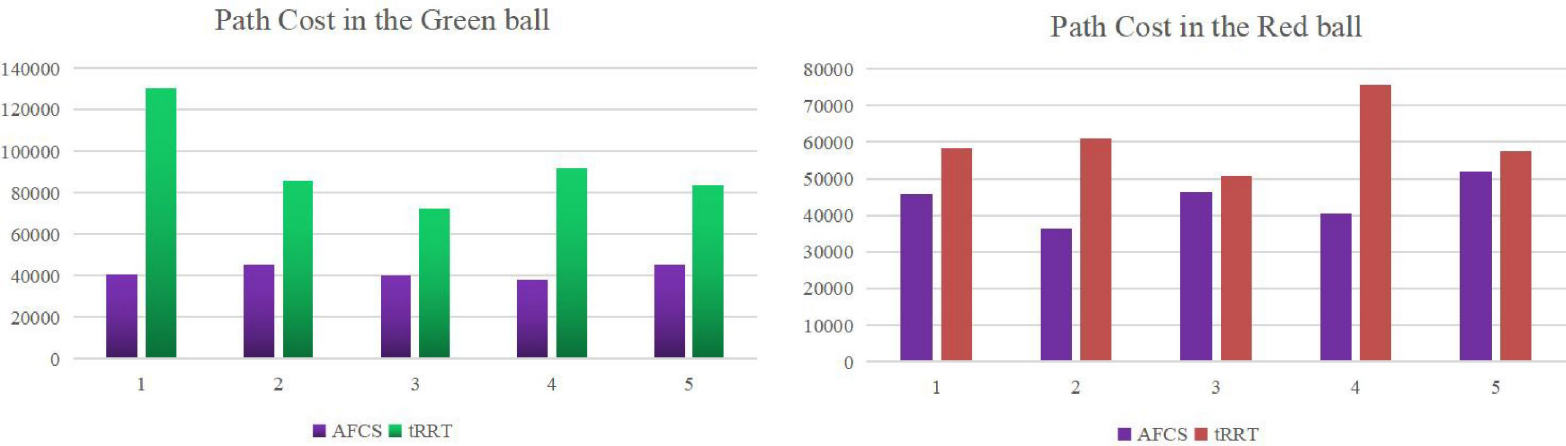
The path cost obtained of the AFCS algorithm and tRRT algorithm in two environments.

Path quality is an important factor to measure the planning efficiency of the algorithm. Path cost and the number of path nodes are important performances of path quality. In this paper, the sum of distance between nodes on the path is taken as the path cost. In this paper, the path cost obtained by running the AFCS algorithm and tRRT algorithm 5 times in two environments is shown in Figure 15.

In Figure 15, the path cost of the AFCS algorithm is about 20% smaller than that of the tRRT algorithm in the red sphere environment. In a more complex environment (green sphere environment), the path cost of the adaptive field co-sampling algorithm is about 50% less than that of the tRRT algorithm, which indicates that the path of the adaptive field co-sampling algorithm is shorter. After running the algorithm 5 times, the path cost float of the AFCS algorithm is smaller than that of the tRRT algorithm, which indicates that the AFCS algorithm has better stability.

**Figure 16:**
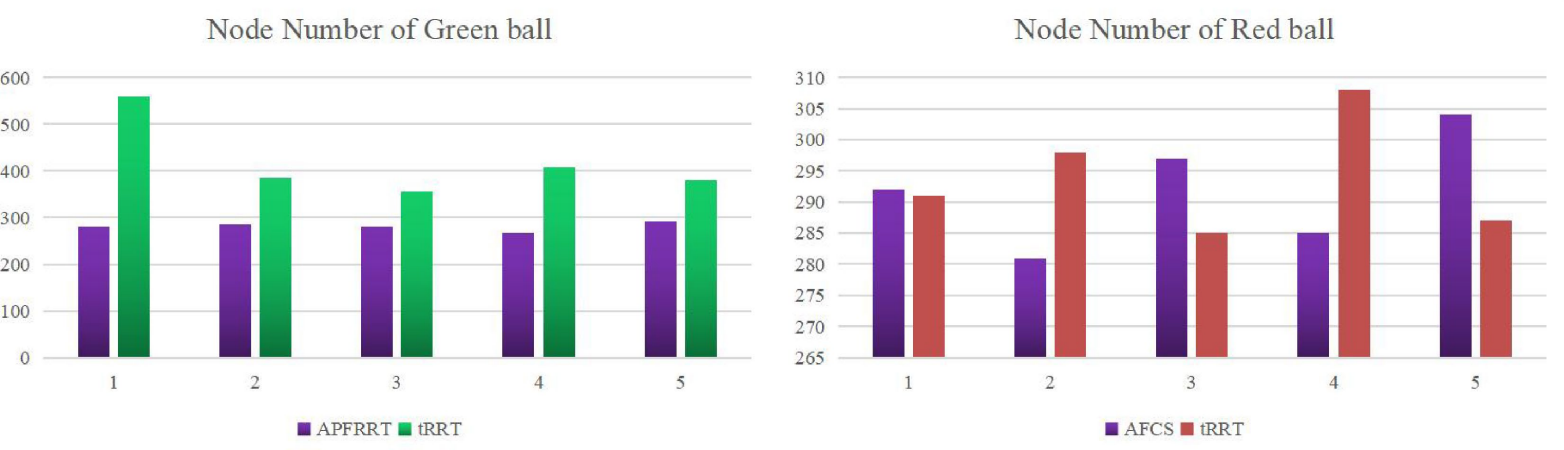
The number of path nodes of the AFCS algorithm and tRRT algorithm.

In this paper, the AFCS algorithm and tRRT algorithm were run five times, respectively, in two environments, and the number of path nodes was obtained, as shown in Figure 16.

In Figure 16, the number of path nodes planned by the AFCS algorithm for five times is also smaller than that of the tRRT algorithm in the red sphere environment, and the number of nodes decreases within a range of 2–5%. In the green sphere environment, the advantage of the adaptive field co-sampling algorithm is more obvious, and the reduction interval of the number of path nodes for 5 times is 21.1–50%. For different complex environments, the number of path nodes of the AFCS algorithm fluctuates little, ranging from 1.4 to 5.4%.

### 3.2 Experiment of industrial robot

**Figure 17:**
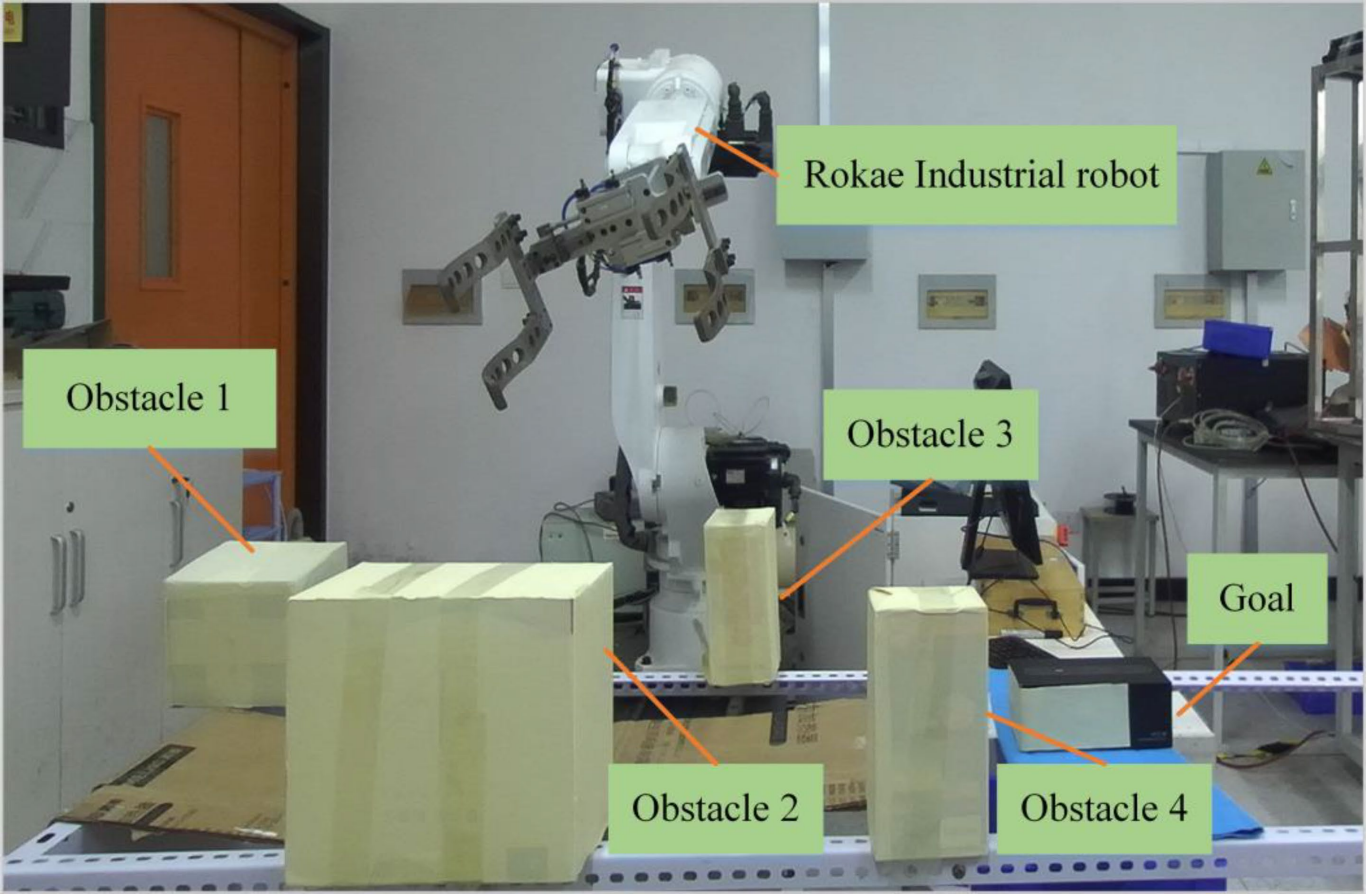
The communication and logical relationship of the three hardware devices.

In order to verify the practicability of the algorithm, the path planning simulation experiment is carried out combining the AFCS algorithm with ROAKE industrial robot XB20 in the green sphere environment. The whole system of the path planning experiment is composed of computer, ROKAE industrial robot XB20 and ZED2 depth camera. Among them, the communication and logical relationship of the three hardware devices are shown in Figure 17.

**Figure 18:**
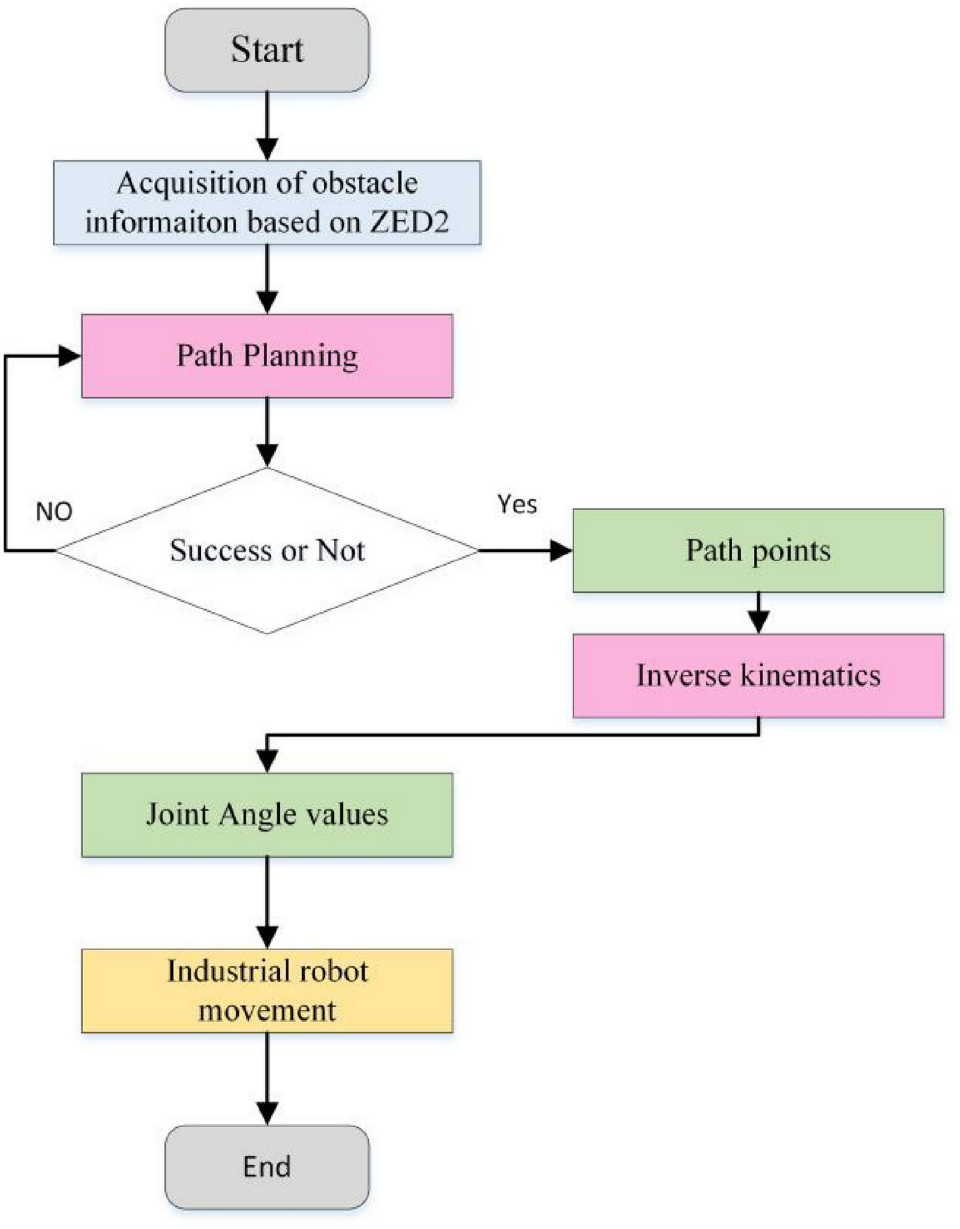
The flow chart of the whole experiment.

Based on the above hardware devices, the experimental flow is shown in Figure 18. The whole process of the experiment consisted of three parts (as shown in Figure 18). The first one is obstacle recognition based on ZED2 depth camera, which mainly obtains the position of obstacles for path planning of industrial robots. The second is path planning based on adaptive field cooperation, which mainly obtains collision-free paths of industrial robots. The third is to control the operation of industrial robots based on collision-free paths (Table 4).

**Table 4:** The result of calibration of binocular camera.

Category of parameters	Parameters
Intrinsic parameter of left camera	$f_{x}$ =21.853, $f_{y}$ =20.206, $c_{x}$ =22.023, $c_{y}$ =612.37
Radial distortion parameters of the left camera	$k_{1}$ = − 0.0108, $k_{2}$ = 0.3905, $k_{3}$ = − 0.9366
Tangential distortion parameters of the left camera	$p_{1}$ = − 0.0257, $p_{2}$ = 0.0113
Intrinsic parameter of right camera	$f_{x}$ =21.234, $f_{y}$ =21.379, $c_{x}$ =21.666, $c_{y}$ =609.82
Radial distortion parameters of the left camera	$k_{1}$ = 0.0149, $k_{2}$ = 0.0693, $k_{3}$ = − 0.1083
Tangential distortion parameters of the right camera	$p_{1}$ = − 0.0210, $p_{2}$ = 0.0135
External parameters of binocular camera	R =[0.9906 0.0053 0.0121 −0.0055 0.9889 0.0002 −0.0131 -0.0103 0.9809] T =[−191.2268–6.7915 -31.7064]

Specifically, the experimental steps and the results of key steps are as follows:

1. The information of the experiment scene can be obtained by the depth camera. And the 3D coordinates of obstacles in the industrial robot coordinate system can be obtained by binocular camera calibration and hand-eye calibration.

The results of hand-eye calibration are shown in Equation 18:


$$X=[\begin{array}{cccc} 0.9976 & -0.0196 & -0.0668 & 16.7035\\ 0.0222 & 0.9990 & 0.0389 & 6.4285\\ 0.0660 & -0.0403 & 0.9970 & 108.3157\\ 0 & 0 & 0 & 1.0000 \end{array}]$$ (18)

1. Based on the three-dimensional coordinates of obstacles, the path planning is carried out by the adaptive field co-sampling algorithm, and the Angle values of each joint of the industrial robot are obtained based on the inverse kinematics solution method.
2. The time series of six joint angles of industrial robot is obtained by using the trajectory planning algorithm (7th degree polynomial trajectory planning algorithm).
3. Socket software is used to send the joint Angle value of the industrial robot to ROKAE industrial robot XB20 to control the movement of it.

**Figure 19:**
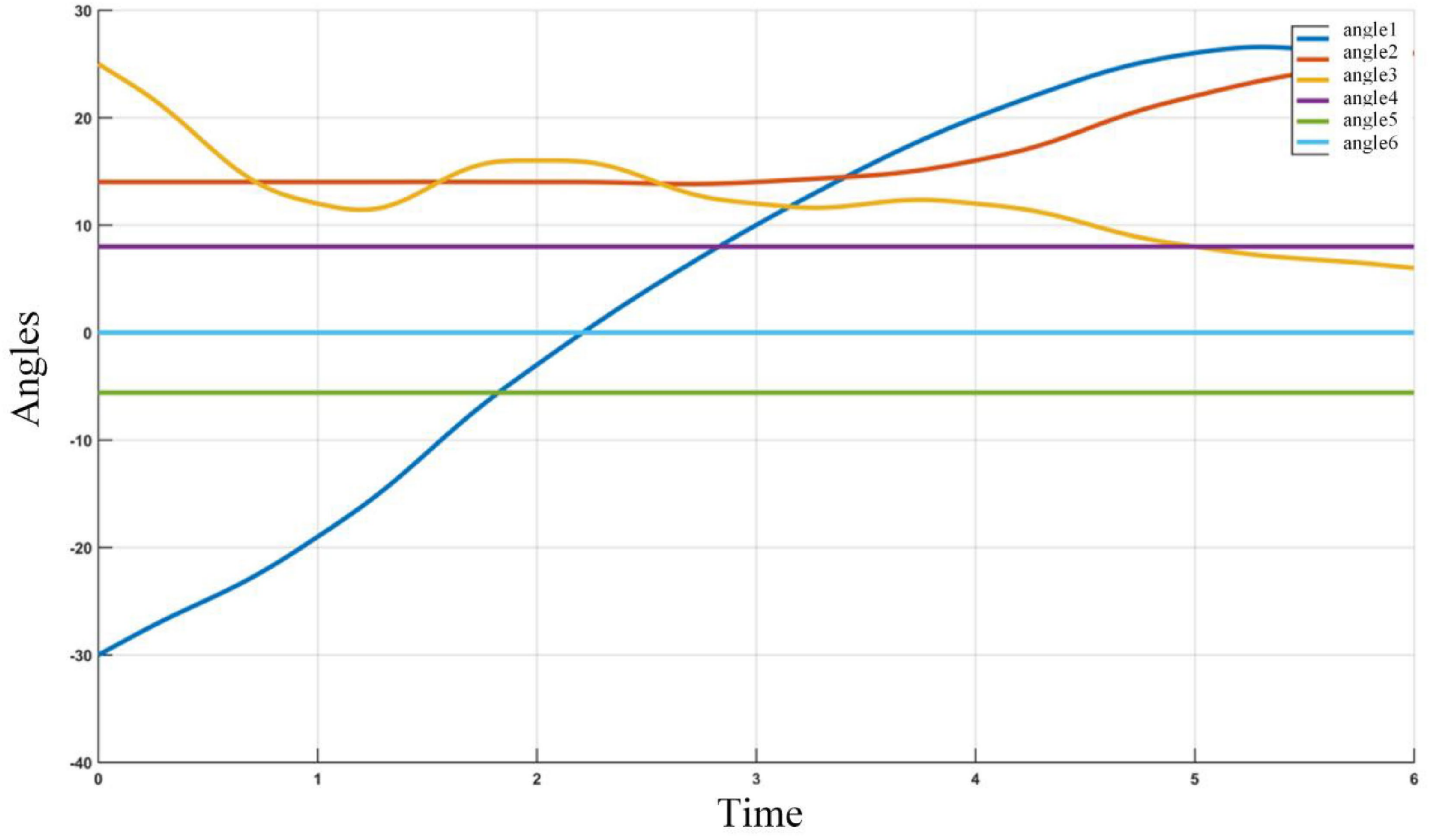
The angle changes of the six joints of ROKAE industrial robot XB20.

During the planning process, the Angle changes of the six joints of ROKAE industrial robot XB20 are shown in Figure 19.

**Figure 20:**
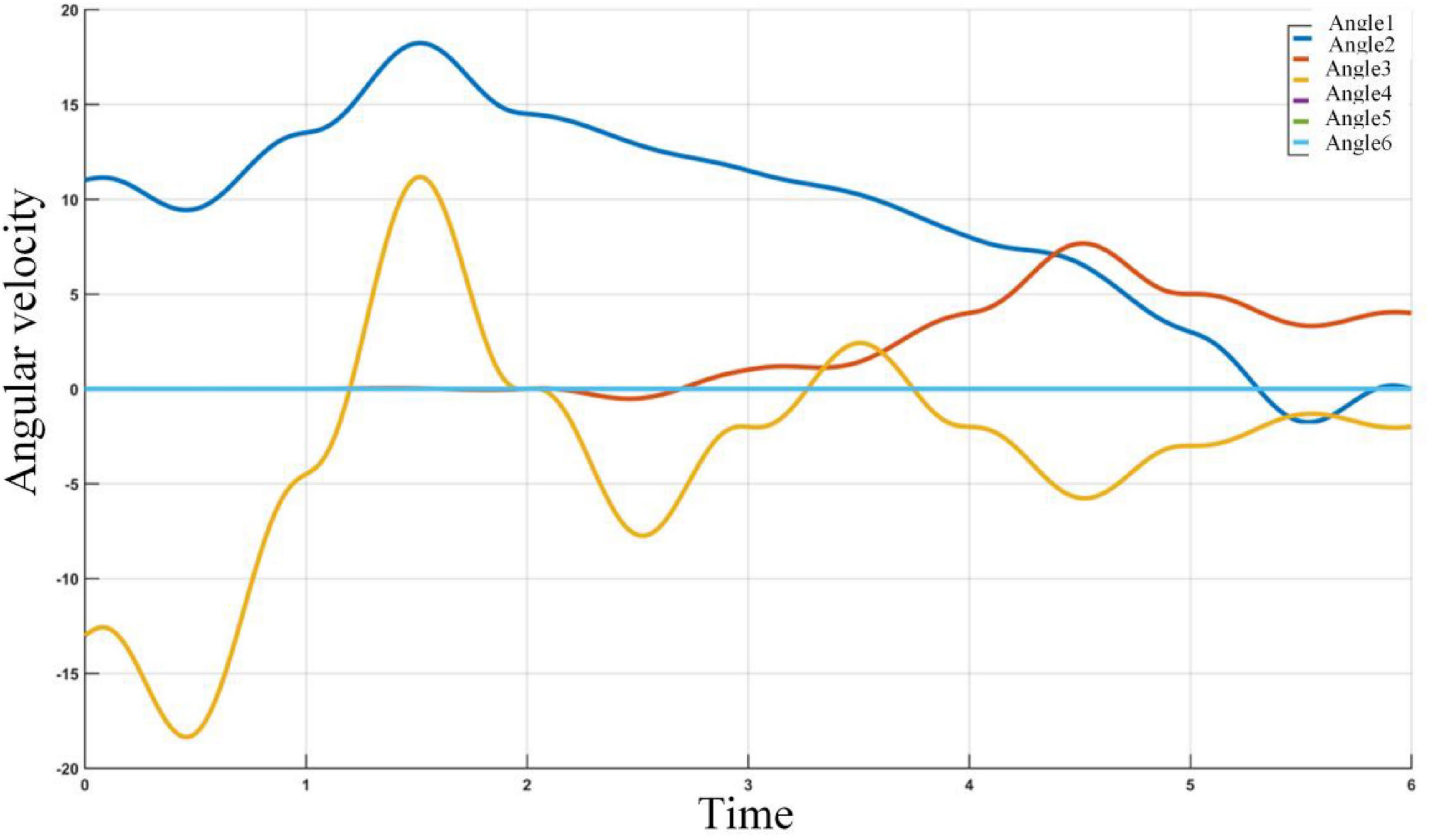
The joint Angle velocity curve of 6 joints of path planning based on adaptive field cooperative sampling path planning algorithm.

The joint Angle velocity curve of 6 joints of path planning based on adaptive field cooperative sampling path planning algorithm is shown in the Figure 20.

**Figure 21:**
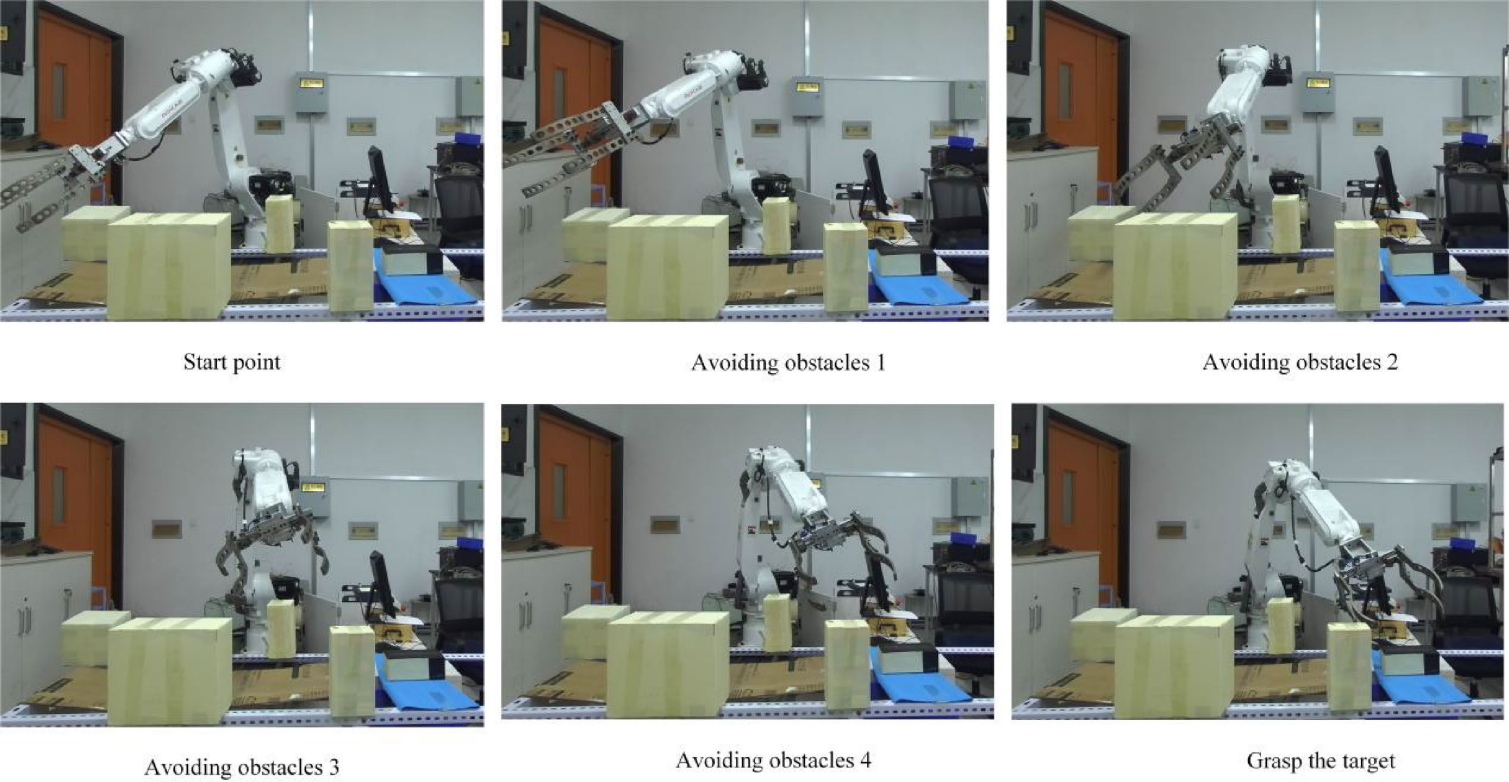
The process of path planning of industrial robots based on adaptive field cooperative sampling algorithm.

It can be seen from the change curve of joint Angle value of industrial robots in Figure 20, the adaptive field cooperative sampling path planning algorithm can drive industrial robots to obtain a collision-free smooth path. The process of path planning of industrial robots based on adaptive field cooperative sampling algorithm is shown in Figure 21.

## 4 Conclusion

This article studies the path planning of industrial robots from the unique perspective of improving the intelligence of path planning algorithm. And an AFCS algorithm with strong environmental adaptability is proposed. It uses the traditional RRT algorithm as the main framework to realize path planning. For the disadvantages of redundant sampling and low efficiency of the traditional RRT algorithm, this paper designs an optimal sampling strategy and improves the node expansion stage. The optimal sampling strategy not only solves the problem of redundant sampling, but also improves the quality of sampling points. The optimal sampling strategy is also beneficial to improve the path optimality. In the expansion stage, this paper firstly improves the traditional APF algorithm, and then introduces it to the node expansion stage of the traditional RRT algorithm. This approach provides a theoretical basis for the generation of new nodes and improves the overall efficiency of the algorithm. More importantly, compared with other algorithms, the efficiency and practicability of the AFCS algorithm are improved, and the adaptability to the environment is significantly improved in the path planning of industrial robots. This approach not only provides ideas for the intelligent development of path planning algorithms, but also provides guarantees for the intelligent development of industrial robots.

## Data Availability

The raw data supporting the conclusions of this article will be made available by the authors, without undue reservation.
