# Correction: Path planning of industrial robots based on the adaptive field cooperative sampling algorithm

**DOI:** 10.3389/fnbot.2025.1754834

**Published:** 2025-12-18

**Authors:** Yongbo Zhuang, Sha Luo, Qingdang Li, Dianming Chu, Wenjuan Bai, Xintao Liu, Mingyuan Fan, Lv Wei

**Affiliations:** 1 College of Electromechanical Engineering, Qingdao University of Science and Technology, Shandong Province, China; 2 Shandong Gaomi Technician Institute, Shandong Province, China

**Keywords:** industrial robot, path planning, RRT, APF, AFCs

In the published article, the equal contribution symbol and phrase “^†^These authors share first authorship” were erroneously included for Yongbo Zhuang and Sha Luo in the author list. This symbol and phrase have now been removed, with Yongbo Zhuang remaining the sole first author.

The original version of this article has been updated.

